# Plant Immune Memory in Systemic Tissue Does Not Involve Changes in Rapid Calcium Signaling

**DOI:** 10.3389/fpls.2021.798230

**Published:** 2021-12-14

**Authors:** Bernadette Eichstädt, Sarah Lederer, Fabian Trempel, Xiyuan Jiang, Tiziana Guerra, Rainer Waadt, Justin Lee, Anja Liese, Tina Romeis

**Affiliations:** ^1^Dahlem Centre of Plant Sciences, Freie Universität Berlin, Berlin, Germany; ^2^Department for Biochemistry of Plant Interactions, Leibniz Institute of Plant Biochemistry, Halle (Saale), Germany; ^3^Leibniz Institute of Vegetable and Ornamental Crops, Großbeeren, Germany; ^4^Entwicklungsbiologie der Pflanzen, Centre for Organismal Studies, Ruprecht-Karls-Universität Heidelberg, Heidelberg, Germany; ^5^Institut für Biologie und Biotechnologie der Pflanzen, Westfälische Wilhelms-Universität Münster, Münster, Germany

**Keywords:** calcium signature, systemic signaling, priming, R-GECO1, CPK5

## Abstract

Upon pathogen recognition, a transient rise in cytoplasmic calcium levels is one of the earliest events in plants and a prerequisite for defense initiation and signal propagation from a local site to systemic plant tissues. However, it is unclear if calcium signaling differs in the context of priming: Do plants exposed to a first pathogen stimulus and have consequently established systemic acquired resistance (SAR) display altered calcium responses to a second pathogen stimulus? Several calcium indicator systems including aequorin, YC3.6 or R-GECO1 have been used to document local calcium responses to the bacterial flg22 peptide but systemic calcium imaging within a single plant remains a technical challenge. Here, we report on an experimental approach to monitor flg22-induced calcium responses in systemic leaves of primed plants. The calcium-dependent protein kinase CPK5 is a key calcium sensor and regulator of the NADPH oxidase RBOHD and plays a role in the systemic calcium-ROS signal propagation. We therefore compared flg22-induced cytoplasmic calcium changes in *Arabidopsis* wild-type, *cpk5* mutant and CPK5-overexpressing plants (exhibiting constitutive priming) by introgressing the calcium indicator R-GECO1-mTurquoise that allows internal normalization through mTurquoise fluorescence. Aequorin-based analyses were included for comparison. Based on the R-GECO1-mTurquoise data, CPK5-OE appears to reinforce an “oscillatory-like” Ca^2+^ signature in flg22-treated local tissues. However, no change was observed in the flg22-induced calcium response in the systemic tissues of plants that had been pre-challenged by a priming stimulus – neither in wild-type nor in *cpk5* or CPK5-OE-lines. These data indicate that the mechanistic manifestation of a plant immune memory in distal plant parts required for enhanced pathogen resistance does not include changes in rapid calcium signaling upstream of CPK5 but rather relies on downstream defense responses.

## Introduction

Plants that experience a primary attack by microbial pathogens not only induce rapid local immune responses but are also able to build a more long-term systemic immune memory, the so-called systemic acquired resistance (SAR). Primed plants are sensitized toward a secondary infection and often induce faster and stronger immune reactions that ultimately impede pathogen growth. Defense priming requires an initial recognition of a pathogen (or conserved microbe-derived molecules termed pathogen-associated molecular patterns, PAMPs), the initiation of local immune reactions, a defense signal propagation from local to distal sites of the plant (systemic tissues), and the onset and maintenance of SAR involving phytohormone-dependent changes in gene expression and defense metabolite synthesis (Hake and Romeis, 2019; Hilker and Schmulling, 2019).

A transient change of the cytoplasmic calcium concentration ([Ca^2+^]_cyt_) is one of the earliest events in plant cells upon pathogen recognition, and many studies applying different calcium indicators reported on distinct [Ca^2+^]_cyt_ patterns in response to PAMPs, e.g., bacterial flg22 (Ranf et al., 2011; Maintz et al., 2014; Seybold et al., 2014; Thor and Peiter, 2014; Keinath et al., 2015; Aldon et al., 2018; Tian et al., 2020). Furthermore, calcium and calcium signaling being instrumental during defense signal propagation from a local attacked site to systemic parts of a plant has been observed as a spread of a calcium signals through the plant along the vasculature and beyond (Romeis and Herde, 2014; Kiep et al., 2015; Nguyen et al., 2018; Toyota et al., 2018; Shao et al., 2020; Fichman and Mittler, 2021). However, it is unknown whether [Ca^2+^]_cyt_ changes contribute to SAR. Defense priming of SAR is mechanistically correlated to changes in gene expression including accumulation of the master transcription factor *SARD1*, on maintaining phytohormone salicylic acid (SA)-based transcriptional reprogramming, or on epigenetic modifications. But it is unclear whether and how calcium signaling contributes to the mechanism necessary to acquire an immune memory. Do plants that have experienced a first priming pathogen stimulus and have consequently established SAR display a different [Ca^2+^]_cyt_ change pattern in response to perception and recognition of a second triggering pathogen stimulus?

Studies on immune-related [Ca^2+^]_cyt_ changes have been performed in cell cultures, protoplasts, young seedlings, excised leaf disks or in epidermal peels with a focus on single guard cells. These were often investigated in response to direct pathogen contact or to purified PAMPs such as flg22, pep13, chitin, or liposaccharides. The degree and patterns of calcium signal changes were recorded over time employing genetically encoded calcium indicator (GECI) systems such as aequorin, cameleon YC3.6 or more recently GCaMP/R-GECO1 and their derived variants (Blume et al., 2000; Kwaaitaal et al., 2011; Ranf et al., 2011; Thor and Peiter, 2014; Keinath et al., 2015; DeFalco et al., 2017; Hilleary et al., 2020; Li et al., 2021). Also, [Ca^2+^]_cyt_ changes have been employed as a signaling read-out in forward and reverse genetic screens to dissect the roles of various genes in plant defense (Ranf et al., 2011, 2012; Tian et al., 2019; Hilleary et al., 2020; Thor et al., 2020). However, none of these systems have been employed for imaging of systemic calcium responses in the context of SAR. Such investigations need to be conducted in adult plants competent to mount SAR, where a selected leaf is treated by a local priming stimulus, and after a gap in time of 2 days, a distal leaf is exposed to a secondary stimulus, and the consequence in the induced calcium response is recorded. The challenge of such an approach is the fluorescence emission-based calcium imaging, which in principle would have to cover an entire “primed” leaf, but ideally at single cell resolution.

In plant cells, changes in the intracellular [Ca^2+^] are decoded and transduced by calcium sensor proteins and their interaction partners, such as CaMs, CMLs, CBL-CIPKs and CPKs (McCormack et al., 2005; Batistič and Kudla, 2012; Aldon et al., 2018; Kudla et al., 2018; Shi et al., 2018; Bredow and Monaghan, 2019; Mohanta et al., 2019). Among these decoder proteins is the 34 member-containing gene family of calcium-dependent protein kinases (CPK in *Arabidopsis*), in which a calcium sensor and protein kinase effector domain are united within a single molecule (Liese and Romeis, 2013; Schulz et al., 2013; Simeunovic et al., 2016; Yip Delormel and Boudsocq, 2019). Several distinct CPK members have been characterized as positive (CPKs 1,2,4,5,6,11) or negative (CPK28) regulators during the local initiation of PAMP-induced immune responses, subsequent to receptor-mediated pathogen recognition (Boudsocq et al., 2010; Gao et al., 2013; Kadota et al., 2014; Monaghan et al., 2014, 2015; Bredow et al., 2021). Among these, CPK5 is a key signaling hub of immune signaling, which correlates with its unique high affinity for calcium (*K*_*d*_ ∼100 nM) for enzyme activation. This is near the resting [Ca^2+^]_cyt_ of an un-challenged plant cell, thus rendering the enzyme highly responsive to small [Ca^2+^]_cyt_ changes (Guerra et al., 2020). Besides its function in local basal immunity, CPK5 was additionally shown to contribute to a calcium- and NADPH-oxidase RBOHD-mediated calcium/ROS-based auto-propagating mechanism that is assumed to be crucial for the defense signal spread from local to distal parts of a plant (Dubiella et al., 2013; Hake and Romeis, 2019). Furthermore, CPK5 is required for priming of a *SARD1*-dependent systemic, long-term immune memory. Enhanced CPK5 signaling in CPK5-YFP overexpressing lines leads to an increased pathogen resistance status, with the plants displaying constitutive priming, manifested by an increase in *SARD1* gene expression, high levels of the defense metabolite *N*-hydroxy pipecolic acid (NHP), and of the phytohormone SA (Guerra et al., 2020). When challenged with a priming infection by an avirulent bacterial pathogen, and a second triggering infection with a virulent pathogen, these plants exhibited enhanced SAR with an almost complete block of pathogen proliferation (i.e., a display of “super-priming”). In contrast, in a *cpk5,cpk6* double mutant line, the lack of *CPK5* and its close CPK homolog *CPK6* lead to an increase in pathogen susceptibility, and when subjected to a priming and triggering context, plants could not be primed and were unable to mount SAR (Gao et al., 2013; Guerra et al., 2020). CPK5 is a key positive regulator in priming and promoting disease resistance. Here, we aim to address if lines differing in their priming status also display altered stimulus-induced [Ca^2+^]_cyt_ changes upon a second (triggering) stimulus.

The GECI R-GECO1 has been successfully used in live cell imaging to resolve PAMP-triggered calcium transients with a high sensitivity but, due to its intensiometric fluorescence readout, suffered from a [Ca^2+^]_cyt_ calculation bias if GECI levels differ between lines or during time course of experiments (Keinath et al., 2015; Waadt et al., 2017). To investigate systemic calcium responses and compare different lines in the context of priming in adult plants over a time course of 2 days, we chose an improved next-generation R-GECO1-mTurqouise (RGmT) system, where the reporter additionally incorporates as an internal mTurquoise (mT) fluorescent protein reference for normalization and validation of the GECI amount throughout the experiment (Waadt et al., 2017). The RGmT sensor was introduced into the CPK5-overexpression line *CPK5#7* and into *cpk5, rbohD, and fls2* mutants by crossing. We found that under conditions of enhanced CPK5 signaling, flg22 induces a more pronounced pattern into distinct peaks of [Ca^2+^]_cyt_ changes in local tissue. By contrast, in a systemic leaf, the overall calcium pattern (signal amplitude, form, and timing) upon flg22 stimulation did not significantly differ between plants of different priming status – either through pre-exposure to flg22 priming stimulus, “*super-priming*” (through overexpressing *CPK5*) or reduced priming (*cpk5* background). These data indicate that the systemic immune signaling causal to enhanced pathogen resistance in SAR does not employ alterations in the early [Ca^2+^]_cyt_ change but relies on downstream defense processes.

## Materials and Methods

### Plant Material and Growth Conditions

*Arabidopsis thaliana* (Col-0) was used throughout this study. Seeds were either sown on 0.5 x Murashige and Skoog (MS) media containing 500 mg/L MES and vitamins (Duchefa, Netherlands), 1% [w/v] sucrose, 0.8% [w/v] phytoagar (Duchefa), pH 5.7 adjusted with KOH, or grown in soil. Seeds were stratified for 2 days at 4°C, and after transfer to individual pots or jiffy-7 soil (Jiffy Products, Norway), the plants were maintained under short-day conditions (8 h day light, 150 μE, 20 – 24°C; 60% RH). Seedlings carrying constructs with fluorophores were screened for fluorescence of the respective fluorescence protein using a fluorescence stereo zoom microscope (Zeiss Axio Zoom.V16, Zeiss, Germany) before the transfer to individual pots. Plants were grown for a total of 6 weeks under short day condition. For aequorin-based calcium assays, seeds were first surface sterilized and stratified for 2 days at 4°C and grown in liquid MS medium for 8–10 days under long day conditions (16 h light, 8 h dark, 21°C). Flg22 and an inactive flagellin variant from *Agrobacterium tumefaciens* were synthesized on an in-house peptide synthesizer and used as previously described (Ranf et al., 2011).

### Generation of Transgenic Plants

For generating transgenic *A. thaliana* lines carrying R-GECO1-mTurquoise (RGmT) Colombia-0 wildtype plants were transformed by floral dip method. Plasmid pGGZ-RW253 (pUBQ10-N-decoy-R-GECO1-GSL-mTurquoise-tHSP18.2M-HygR) was generated *via* GreenGate cloning using previously published modules (Lampropoulos et al., 2013; Waadt et al., 2017). Seeds of homozygous *RGmT* lines were harvested and crossed with the mutants *cpk5* (SAIL_657C06), *rbohD* (SALK_070610), and *fls2* (SALK_062054) and the CPK5-overexpressing line *CPK5#7* that have been described before (Ranf et al., 2012; Dubiella et al., 2013). The generated lines were selected for BASTA (phosphinothricin, glufosinate ammonium) or hygromycin B resistance and fluorescence to obtain independent transformants. For aequorin luminescence measurements, wildtype plants expressing cytosolic p35S-apoaequorin (pMAQ2) were used (Knight et al., 1991). Seeds of a homozygous line were used to cross with the *cpk5* (SAIL_657C06) and the CPK5-overexpressing line *CPK5#7*.

### Calcium Measurements

#### R-GECO1 Based Calcium Imaging

##### Local Calcium Imaging

For sample preparation, 2 well chambered coverslips (IBIDI, Germany) were coated with a thin layer of medical adhesive (Ulrich Swiss, Switzerland). After 10 min of evaporation of volatile medical adhesive components excess medical adhesive was removed by washing three times with water. The leaf abaxial epidermis was then glued to the medical adhesive layer, and magic tape (Scotch, Germany) was taped to the adaxial epidermis and pulled away. Mesophyll cell layers were then removed carefully by affixing and pulling away with magic tape, leaving a single-layer of lower epidermis behind that was attached to the IBIDI slide. Immediately the coverslip was immersed with 1 ml plant imaging buffer (10 mM MES-Tris pH 5.6, 5 mM KCl, 50 μM CaCl_2_) and the samples were incubated for recovery in a phyto chamber or growth cabinet overnight. The samples were incubated under light at 22–24°C for at least 1 h before imaging. Confocal laser scanning microscopy was performed in bottom imaging mode on a Zeiss LSM 780 or 880 system (Zeiss) using a 40 × water immersion objective (LD C-APOCHROME, 40 x/1.1 Korr UV-VIS M27; Zeiss) at a zoom factor of 0.8. 16-bit images were acquired every 4 s with a frame size of 512 × 512 pixels and a pinhole of 200. mTurquoise and R-GECO1 were excited with 458 or 561 nm, respectively and an emission-range between 470 and 540 nm for mTurquoise or 590 and 640 nm for R-GECO1 was used for detection. Gain was set to 850 and laser intensity settings were adjusted individually to have comparable baseline intensity values for each experiment. For flg22 treatments, 50-fold and for ATP treatments 10-fold concentrations of the respective agent were prepared in water and added in a 1:50 or 1:10 volume ratio to the imaging chamber to avoid sample movement. The image processing was performed using Fiji (Schindelin et al., 2012). Images acquired in Zen Z2.3 SP1 FP3 black (Zeiss) were imported as single channel files into Fiji and all following steps were conducted for both fluorescence channels (R-GECO1 and mTurquoise): Gaussian blur filter set to 1, conversion into 32-bit, threshold adjustment (stack histogram) and selection of an appropriate look-up table. For further analyses, 26 regions of interest were selected and imported into the ROI manger. ROI_1 included the whole image. For ROIs 2–26 the image of 512 × 512 pixel was divided into 25 ROIs of 102.4 × 102.4 pixels each (Supplementary Figure 6A). The mean gray values of these ROIs were used for further calculations. The RGmT ratio was calculated *via* dividing R-GECO1 mean gray values over mTurquoise mean gray values. The resulting RGmT emission ratio was normalized to the mean of the 10 min (flg22) or 5 min (ATP) base line ratio for each ROI before flg22 or ATP treatment (R/R_0_). For each ROI the following parameters were analyzed: maximal signal change after treatment; time until maximal signal change; number of local maxima, and time between first two local maxima. ROI_1 and the selected ROI_S_ with the highest number of local maxima were chosen for comparison of different genotypes. The graphs of single ROIs were generated in R, using R Studio (PBC, United States) incl. the packages tidyverse and reshape2 (Supplementary Figure 6; Wickham, 2007; Wickham et al., 2019; R Core Team, 2021). Scripts for the ImageJ macro and analysis in R are available on request.

##### Analysis of Resting [Ca^2+^]_cyt_ Levels

We monitored R-GECO1 and mT fluorescence without stress application in leaf disks to compare resting [Ca^2+^]_cyt_ levels between RGmT crosses. Leaf disks were sampled from 6-week-old stable *A. thaliana* expression lines and transferred to 1 mL distilled water. The sampled plants were transferred back to prior growth conditions for an overnight recovery period to minimize calcium signals induced by wounding. Leaf samples were imaged with a fluorescence stereo zoom microscope (Zeiss Axio Zoom.V16, Zeiss) using a 1 × objective and a zoom factor of 10. Optical filters for mTurquoise (λ_excitation_ 436/20 nm, λ_emission_ 480/40 nm) with an exposure time of 1.5 s and for R-GECO1 (λ_excitation_ 550/25 nm, λ_emission_ 605/70 nm) exposure time of 1.8 s were used for fluorescence detection. For image processing the following steps were conducted for R-GECO1 and mT channels using Fiji: Gaussian blur filter set to 1, conversion to 32 bit, threshold adjustment (stack histogram) and the mean gray values of the whole image area were used for calculating fluorescence intensities. Analyses revealed higher signal intensities for R-GECO1 and mTurquoise channels in the *CPK5#7* crossed line, probably indicating a higher protein abundance (Supplementary Figure 2A). However, the denominator channel mTurquoise exhibited an even higher increased fluorescence intensity compared to other RGmT crosses than R-GECO1 channel, and as consequence, the ratiometric readout fluorescence_R–GECO1_/fluorescence_mTurquoise_ was decreased, indicating apparent lower resting [Ca^2+^]_cyt_ levels. To test if altered fluorescence ratios of R-GECO1/mTurquoise may rely in a high sensor protein amount, we transiently expressed and imaged increasing RGmT protein concentrations in the same *N. benthamania* leaf. Transient expression in *Nicotiana benthamiana* was conducted as described in Franz et al. (2011). For *N. benthamiana* infiltration reciprocal dilutions of *A. tumefaciens* cultures harboring RGmT plasmid and *A. tumefaciens* cultures harboring a plasmid coding for the N-terminal domain of slow anion channel 1 (SLAC1) (Geiger et al., 2010) were mixed leading to OD_600_ concentration gradients of RGmT *A. tumefaciens* cultures from 0.1 to 0.5. The combinatory OD_600_ of *A. tumefaciens* cultures harboring RGmT or SLAC1-NT were kept constant at 0.5. Leaf disks were harvest 3 days after transfection of *N. benthamiana*. Preparation and imaging setup was identical to resting [Ca^2+^]_cyt_ measurements in *A. thaliana*.

##### Systemic Calcium Imaging

Three fully developed “local” leaves of 6-week-old plants were infiltrated with either mock-treatment (10 mM MgCl_2_) or 200 nM flg22 as priming-stimulus using a needleless syringe. After 48 h, a 4 mm diameter leaf disk was harvested from a systemic leaf and fixed adaxial on a 8 well chambered coverslip (IBIDI) using medical adhesive (Ulrich Swiss) and immersed with distilled water. Selection of local and systemic leaves as described in Dubiella et al. (2013). The sample was transferred back to prior growth condition and incubated overnight to minimize calcium signals induced by wounding. The next day calcium imaging of the sample was conducted on the upright fluorescence stereomicroscope Leica M165 FC and M205 FA (Leica, Germany) using a 2x/0.04 objective and a zoom factor of 0.83. During in planta imaging, single RGB images (frame size of all images: 1920 × 1440 pixels) of the R-GECO1 (λEx = 546/10 nm, λEm = 605/70 nm, exposure time 1.5 s) and mTurquoise channels (λEm = 436/20 nm λEm = 480/40 nm, exposure time 1.5 s) were acquired. In the following experiment to enable, a high imaging frequency only R-GECO1 was detected every 4 s over 42 min. This step was required due to the technical limitations of the microscopes fluorescence filter system. After 2 min 10-fold concentrations of flg22 (200 nM final conc.) prepared in water was added in a 1:10 volume ratio to the imaging chamber as triggering-stimulus to avoid sample movement. Finally, another single image with both channels was recorded. Images acquired during the calcium assay in LAS X (Leica application software) were imported into Fiji and all following steps were conducted for the R-GECO1 channel: Conversion to 8-bit; brightness and contrast adjustment, conversion into 32-bit, threshold adjustment (stack histogram) and selection of 16 colors look-up table. R-GECO1 fluorescence during the time course was normalized to R-GECO1 fluorescence at 2 min (flg22-application; fluorescence time point t_0_ = F_0_) *via* dividing each of the following created 600 images over the R-GECO1 signal at time point t_0_ (F/F_0_) using the image calculator tool in Fiji. The resulting mean gray value was used for further calculations. The RGmT ratio in the beginning and at the end of the assay was calculated *via* dividing R-GECO1 mean gray values by mTurquoise mean gray values using the acquired raw data.

#### Aequorin-Based Calcium Assays

Individual 8–10-day-old seedlings were transferred into 96-well plates, reconstituted with 10 μM coelenterazine and cytosolic calcium measurements were performed as described (Trempel et al., 2016). The calculation for intracellular calcium concentrations is based on the previously described equation: pCa = 0.332588 (−log k) + 5.5593 (where *k* = L/L_max_ i.e., luminescence counts per s/total remaining luminescence counts) (Rentel and Knight, 2004). To facilitate visualization of small differences, the values for relative calcium concentrations (L/L_max_) are shown. An aequorin-expressing line in the *rbohD* background (Ranf et al., 2011) served as a control for the lack of a second flg22-induced calcium peak.

### Western Blotting and Immunodetection

Leaf material was homogenized in liquid nitrogen in a laboratory mill and incubated with a 2:1 weight ratio of 2x SDS sample buffer (60 mM Tris, pH 6.8; 100 mM DTT; 10% (v/v) glycerol; 2% (w/v) SDS; 0.004% (w/v) bromophenol blue) for 5 min at 95°C for subsequent SDS-Page western blotting and immunodetection. For RGmT detection the α-RFP (Rockland, United States) primary antibody was used. For visualization a HRP-conjugated secondary α-rabbit-HRP antibody (Sigma-Aldrich, United States) was applied. Blots were developed using enhanced chemiluminescence (Thermo Scientific, United States) detection.

### Gene Expression Analyses (RT-qPCR)

RNA, isolated with Trizol reagent, was treated with DNAseI (to remove any remaining DNA contamination) and cDNA was synthesized using the RevertAid kit (Thermo Scientific). For realtime quantitative PCR (qPCR), amplicons from cDNA samples were analyzed on an Mx3005P qPCR system (Agilent, United States) after amplification with Maxima SYBR Green qPCR Master Mixes (Thermo Scientific). Relative gene expression values of the genes-of-interest were calculated with the comparative CT method (Schmittgen and Livak, 2008) using the reference gene PP2A [AT1G13320; (Czechowski et al., 2005)]. Primers are listed in Supplementary Table 1.

## Results

### Generation and Characterization of R-GECO-mT Calcium Reporter Lines in *cpk5* and CPK5-OE Lines

**FIGURE 1:**
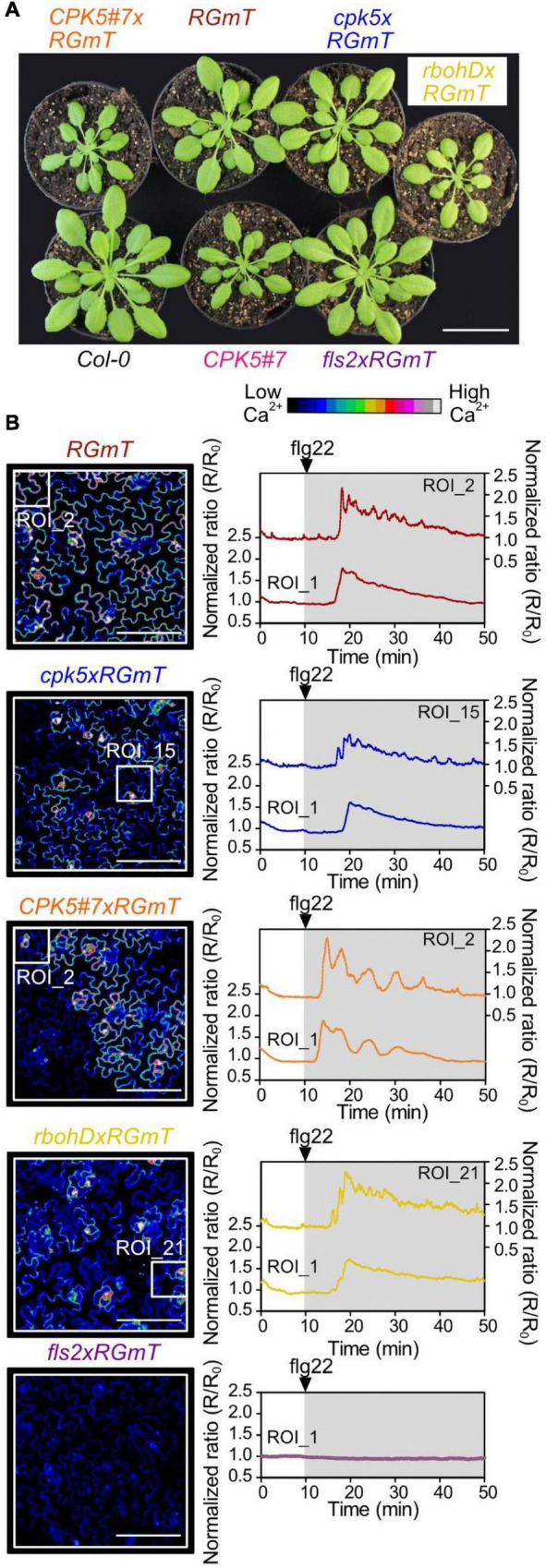
Flg22-induced calcium transients by R-GECO1-mT imaging in epidermal cells of defense signaling mutant- and CPK5 overexpression lines. **(A)** Pictures of 6-week-old plant rosettes of Col-0, line *CPK5#7* overexpressing *CPK5-YFP*, and plants carrying the ratiometric Ca^2+^-sensor R-GECO1-mTurquoise (RGmT) generated by crossing with *CPK5#7*, *cpk5, rbohD*, and *fls2* as indicated. **(B)** Changes in the cytosolic calcium concentration [Ca^2+^]_cyt_ were visualized in response to 200 nM flg22 in epidermal peels of 6 week old plants (left panels) recorded using RGmT for a time period of 40 min after flg22 treatment (right panels). Normalized fluorescence ratios (R/R_0_) over time were calculated from the total acquired image (Region of interest 1 (ROI_1) – left axis) and from a selected ROI (ROI_S_; marked by small squares – right axis) that exhibited the highest number of peaks. Graphs were normalized to mean RGmT ratio of the 10 min before flg22 treatment. The 40 min time interval of recording after the 200 nM flg22 treatment is indicated by the underlying gray area (right panels). Microscopic images represent R-GECO1 fluorescence intensities at the time point of the maximal calcium signal change. Shown are representative experiments with *n* ≥ 6 per analyzed line from three independent sets of plants. Scale bars in panel **(B)** represent 100 μm and 4 cm in panel **(A)**.

To be able to investigate stimulus-induced [Ca^2+^]_cyt_ changes in response flg22 perception, we generated Ca^2+^ reporter lines expressing R-GECO1-mTurquoise (RGmT) in *cpk5* and CPK5-OE (CPK5#7) backgrounds by crossing. Since all GECI lines have been reported to exhibit some level of reduced growth (Waadt et al., 2017), we first characterized the resulting RGmT crosses for any obvious growth phenotype. In 6-week-old plants grown under short day conditions, no obvious defects were observed, except for the slightly smaller plants in the CPK5#7 overexpressing background and in *rbohD*. Both genotypes have already been previously reported to be smaller compared to wild-type (Torres et al., 2002; Dubiella et al., 2013). Importantly, the smaller stature of *CPK5#7* that was previously correlated with a constitutively higher basal resistance compared to wild-type (Dubiella et al., 2013), was not further aggravated through introgression of the RGmT reporter (Figure 1A). Elevated basal expression of the defense marker genes, *PR1* and *NHL10*, characteristic for enhanced CPK5 signaling and a constitutively higher immune status in CPK5-OE, was also confirmed for the *CPK5#7xRGmT* cross (Supplementary Figures 1A,B). In addition, this leads to an enhanced flg22 responsiveness in the CPK5#7 background as is evident from the expression pattern of the flg22-responsive gene, *FRK1* (Supplementary Figure 1C).

To verify comparable expression of the RGmT reporter, both RT-qPCR and immunoblotting were performed where RGmT was shown to be well expressed in all lines. However, the *CPK5#7xRGmT* line expressed about threefold as much RGmT compared to the parental line or RGmT crosses with *cpk5*, *rbohD*, or *fls2* (Supplementary Figures 2A,B). We next compared resting [Ca^2+^]_cyt_ based on R-GECO imaging normalized to mT. Interestingly, an apparently lower F_R–GECO1_/F_mT_ ratio indicative of a lower [Ca^2+^]_cyt_ was observed in *CPK5#7xRGmT* (Supplementary Figure 2C). We thus addressed whether differential levels of the RGmT reporter can distort the [Ca^2+^]_cyt_ calculation. Using the *N. benthamiana* transient expression system, increasing protein levels were independently assessed for R-GECO and mT emissions. The F_R–GECO1_/F_mT_ ratio showed that higher reporter levels indeed generated a lower estimation of the apparent [Ca^2+^]_cyt_ (Supplementary Figures 2D,E) (see the section “Materials and Methods” for further details). This must be taken into consideration for data interpretation of resting [Ca^2+^]_cyt_ when comparing lines of different genetic backgrounds. Despite this caveat, the RGmT reporter is still one of the most suitable systems for imaging stimulus-induced changes in Ca^2+^ transients within systemic tissues of adult plants with high sensitivity.

### Enhanced CPK5 Signaling Triggers Distinct Flg22-Induced Calcium Signatures

Since both, basal and flg22-stimulated defense transcription responses were elevated in the *CPK5#7* plants (Supplementary Figure 1C), we asked if Ca^2+^ signaling responsiveness is also primed. We first focused on the Ca^2+^ response in local tissues exposed to flg22 peptide as a trigger and compared the different lines for stimulus-induced changes in Ca^2+^ transients in epidermal cells of 6-week-old plants. Mathematical models have previously predicted that Ca^2+^ responses reflect the number of measured cells (Dodd et al., 2006), which was proven using R-GECO1-based [Ca^2+^]_cyt_ imaging (Keinath et al., 2015) where the “oscillatory behavior” of the flg22 response was better resolved by focusing on a specific region (i.e., encompassing fewer cells) than an area covering more cells. Hence, we chose similar experimental conditions, where we imaged a region-of-interest (ROI_1) covering 47 ± 17 epidermal cells for a global view and a second smaller ROI (designated as ROI_S_) of 6 ± 2 cells for improved resolution of the Ca^2+^ dynamics (Figure 1B, left panels). ROI_S_ was selected to represent a sub-region with more defined flg22-induced [Ca^2+^]_cyt_ transients (for details of ROI selection see Materials and Methods and Supplementary Figure 6).

**FIGURE 2:**
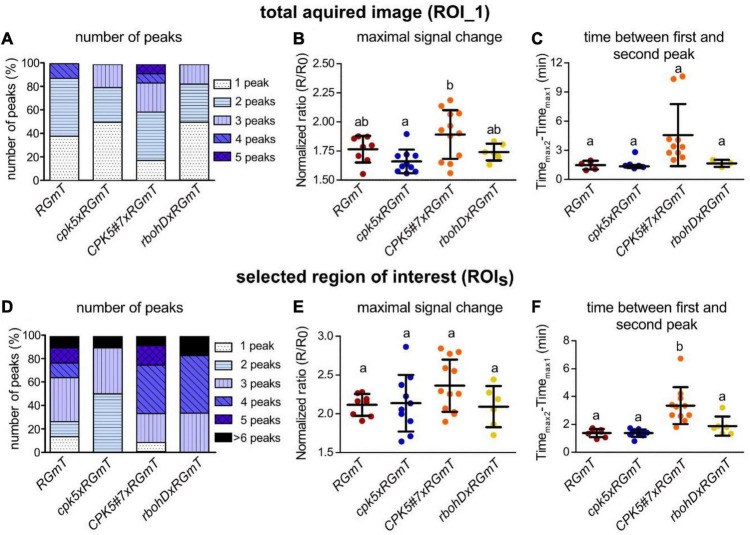
Enhanced CPK5 signaling in line *CPK5#7xRGmT* results in a higher percentage of defined flg22-induced calcium concentration changes (peaks) compared to the wild-type and to *cpk5* or *rbohD* mutant lines. **(A,D)** Number of defined calcium concentration changes (peaks) after treatment with 200 nM flg22 in analyzed genotypes. Shown is the fraction of measurements with the specified number of peaks in percentage. In panels **(B,C,E,F)** dot plots represent maximal signal change and time between the first and second local maximum after flg22 treatment. Shown are means ± SD. Dots represent the individual measurements. Different letters indicate significant differences between the analyzed genotypes [one-way ANOVA and Tukey’s multiple comparisons *post hoc* test (*p* ≤ 0.05)]. For panels **(A,B,D,E)** 6–12 biological replicates per line from three independent sets of plants were analyzed. In panels **(C,F)** measurements with more than one peak were selected [**(C)**, *RGmT*, and *cpk5xRGmT n* = 5, *CPK5#7xRGmT n* = 10, *rbohDxRGmT n* = 3; **(F)**, *RGmT n* = 7, *cpk5xRGmT n* = 10, CPK5#7xRGmT *n* = 11, *rbohDxRGmT n* = 6]. Data from total acquired image (ROI_1) are shown in panels **(A–C)** and from selected ROIs (ROI_S_) in panels **(D–F)**.

Overall, flg22-elicitation induces RGmT responses that are predominantly of a monophasic shape for ROI_1 (i.e., the average of multiple cells) over 40 min, and a more pronounced spiky response for ROI_S_ (Figure 1B, right panels, Supplementary Movie 1). This Ca^2+^ response is specific since no flg22-induced rise in [Ca^2+^]_cyt_ was detected in the *fls2* receptor mutant (Figure 1B, bottom traces). Important to note is that there is substantial variation in the Ca^2+^ response traces between experiments (Supplementary Figure 3), possibly due to asynchronous responses across the tissues, or variations in the perception of the flg22 peptide in the imaged regions. For instance, the lag time between flg22 application to the first Ca^2+^ peak is 9.2 ± 5.8 min (*n* = 36). Here, the large variation in onset of Ca^2+^ signaling may be attributable to technical restrictions related to poor flg22 diffusion/accessibility in the tight space between the leaf surface and the coverslip in the bottom imaging mode. Interestingly, however, traces of the overexpressing *CPK5#7xRGmT* line exhibited a distinct Ca^2+^ signature characterized by defined peaks and longer intervals between the peaks (Figures 1B, 2 and Supplementary Movie 2). To substantiate this perceived difference in [Ca^2+^]_cyt_ changes in *CPK5#7xRGmT*, we consolidated the data of all our RGmT measurements into three parameters defining the Ca^2+^ signature: (1) the number of peaks within the 40 min recording time, (2) the maximum signal change (amplitude), and (3) the time between the first and second peak. A distribution plot showed a shift toward Ca^2+^ traces with an increased “number of peaks” in the *CPK5#7xRGmT* line, which is consistent for both ROI_1 and ROI_S_ (Figures 2A,D). Additionally, the “time between first and second peak” for ROI_S_ (Figure 2F) was significantly enhanced in the *CPK5#7xRGmT* line. Moreover, a statistically significant difference between *CPK5#7xRGmT* compared with *cpk5xRGmT* in the “maximal signal change” for ROI_1 was observed (Figure 2B) with a higher amplitude in the overexpressing and lower amplitude in the corresponding mutant line, respectively. Notably, this apparent boost of the signal amplitude in *CPK5#7xRGmT* or reduction in c*pk5xRGmT* was not seen when ATP was used as a trigger (Supplementary Figure 4A), so that this change in Ca^2+^ signature is elicitor specific. In addition, we could rule out that a somewhat smaller plant size as seen in the CPK5#7 and *rbohD* crosses may lead to different number of cells being imaged within the ROI compared to the other lines, which has been reported to possibly affect the features of a Ca^2+^ response and confound data interpretation (Dodd et al., 2006). However, no correlation between the flg22 response and the number of imaged cells per ROI_1 for the different genotypes was observed (Supplementary Figure 4B).

These data indicate that enhanced immune signaling through overexpression of CPK5 triggers a distinct Ca^2+^ signature in epidermal cells of 6-week-old plants. This can largely be described as increasing number of well-defined peaks with higher amplitude and an increased time-gap between first and second peak.

**FIGURE 3:**
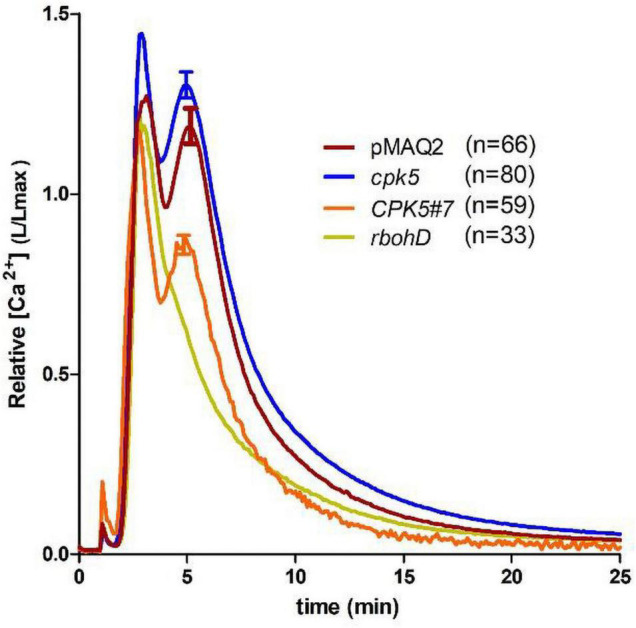
Aequorin-based Ca^2+^ measurements show altered flg22-induced Ca^2+^ response in CPK5-overexpression and *cpk5* mutant lines. Flg22-induced calcium elevations were measured in 8-day-old seedlings. pMAQ2 is the corresponding apoaequorin-expressing parental line used for crosses into the indicated genotypes. Error bars denote standard error of the mean (of the indicated number of seedlings). One-way-ANOVA and Dunnett’s multiple comparisons *post hoc* test (*p* ≤ 0.05) indicate statistical significance to pMAQ2 for all genotypes.

We next compared the flg22-induced Ca^2+^ response using the well-established aequorin-based assay, which has been shown to typically produce a highly reproducible and quantitative response in young seedlings. Therefore, the aequorin-expressing line, pMAQ2, was crossed into *cpk5* and *CPK5#7*. We observed the typical flg22-induced Ca^2+^ change reported for the aequorin system, which comprises of a rapid Ca^2+^increase with twin peaks at 2–3 min and 5–6 min, respectively, and a gradual return to resting [Ca^2+^]_cyt_ within 20–25 min (Figure 3). The overall calcium signature is thus very similar to the monophasic response (ROI_1) recorded with RGmT (Figure 1B). When the previously described aequorin-expressing *rbohD* line (Ranf et al., 2011) was used as a control, the second flg22-induced [Ca^2+^]_cyt_ peak was not detected. This indicates that the second peak is ROS-dependent. The profile of the calcium response (kinetics and twin peak profile) is generally not affected by *cpk5* mutation or *CPK5* overexpression. However, a significant reduction in the Ca^2+^ amplitude in the CPK5#7 line and an increase in the *cpk5* mutant was observed (Figure 3). Taken together, both aequorin and RGmT imaging techniques report an altered Ca^2+^ signature upon a local flg22 stimulus in the CPK5 overexpressing line. These data imply that enhanced CPK5 signaling, correlating with an increase in basal immunity status, display a different Ca^2+^ signature in response to a local PAMP trigger (such as flg22).

### Establishment of an Experimental Set-Up for Systemic Calcium Response Analyses

**FIGURE 4:**
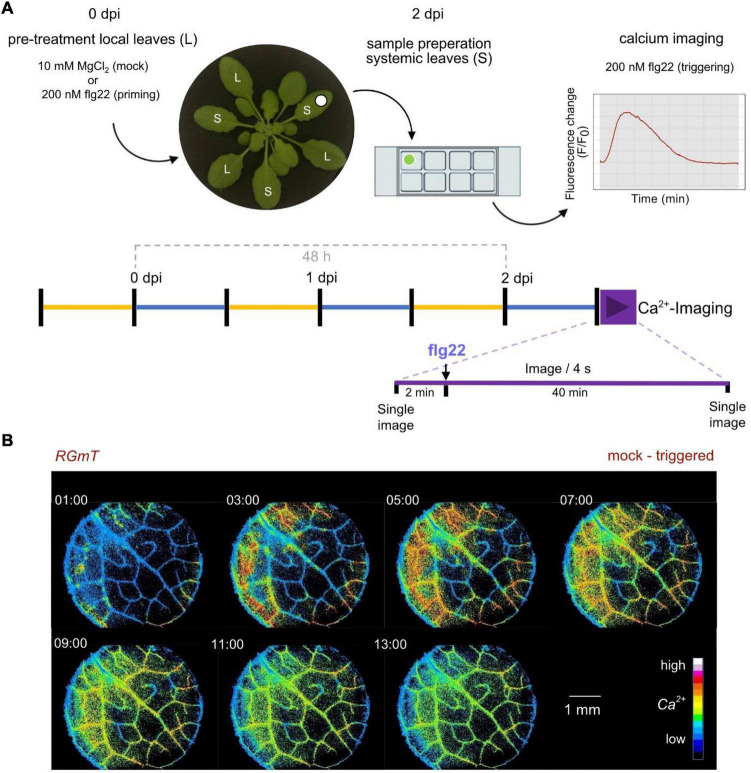
Experimental scheme and imaging analysis of flg22 triggering stimulus-induced calcium changes in systemic leaf tissue disks. **(A)** Experimental scheme of treatment, sampling, and imaging to assess mock-triggered vs. primed-triggered calcium concentration changes. Plants were grown under short day conditions (light periods from 9 am – 5 pm marked in yellow, dark periods marked in blue) over 6 weeks. Three local leaves were infiltrated with mock (10 mM MgCl_2_) or 200 nM flg22 as priming-stimulus (0 dpi). After 2 days post infection (dpi), a systemic leave sample was fixed into a chamber slide. The next morning, samples were transferred to light, 1 h before the measurement, to ensure that each sample received equal amounts of light. In the beginning and at the end of the assay (purple timeline) one single image of each, R-GECO1 and mTurquoise channel was acquired. The R-GECO1 fluorescence was recorded for 42 min with a frame rate of 1 image/4 s. After 2 min, 200 nM flg22 as triggering-stimulus was added to the sample (t_0_). **(B)** Representative image series of flg22 induced calcium concentration changes in a systemic leaf disk. Shown is an example of a mock-triggered sample. The 7 images indicate the increase of [Ca^2+^]_cyt_ in false-colors (16 colors LUT) relative to the time point of flg22 application during the Ca^2+^-Assay (t_0_).

We next investigated whether systemic tissues of plants pre-treated with a first local flg22 trigger, and thus having been “primed” toward an immune memory, display an altered Ca^2+^ response when exposed to a secondary flg22 trigger. As illustrated in the scheme in Figure 4A, three leaves of 6-week-old plants were infiltrated with 200 nM flg22 (or 10 mM MgCl_2_ as a mock control). After 2 days incubation in a growth chamber, a disk from a systemic leaf was sampled and mounted on a microscope chamber slide, incubated again over-night in a growth cabinet for recovery, and incubated for 1 h in light before the second flg22 treatment and RGmT-based calcium imaging. This modification of the experimental setup was necessary because the preparation of epidermal peels (as used in investigation of local responses, Figure 1) can interfere with the priming stimulus in the systemic tissues. The Ca^2+^ response was monitored for 2 min before and 40 min after the flg22 stimulus. We could verify that the mT fluorescence monitored before and after measuring remained uniform for all lines indicating constant RGmT protein level throughout the analysis period. This allowed us to apply an intensiometric quantification for R-GECO1, which simplified data recording at high imaging frequencies. Figure 4B shows the first 13 min after flg22 treatment of an exemplary imaging series, depicting the flg22-induced [Ca^2+^]_cyt_ increase beginning from the edge of the leaf disk and moving toward the middle. This inward movement [Ca^2+^]_cyt_ signals may reflect a gradual access of flg22 from the wounded leaf disk edges or perhaps cell-to-cell Ca^2+^ propagation. Additionally, we also observed occasional local spots of Ca^2+^ increase in some leaf disks but these were not associated to a certain genotype or pre-treatment (see Supplementary Movie 3). Nevertheless, the described system is suitable for monitoring Ca^2+^ responses in systemic tissues of primed plants.

### Flg22-Induced Calcium Response Patterns Are Independent of Priming

**FIGURE 5:**
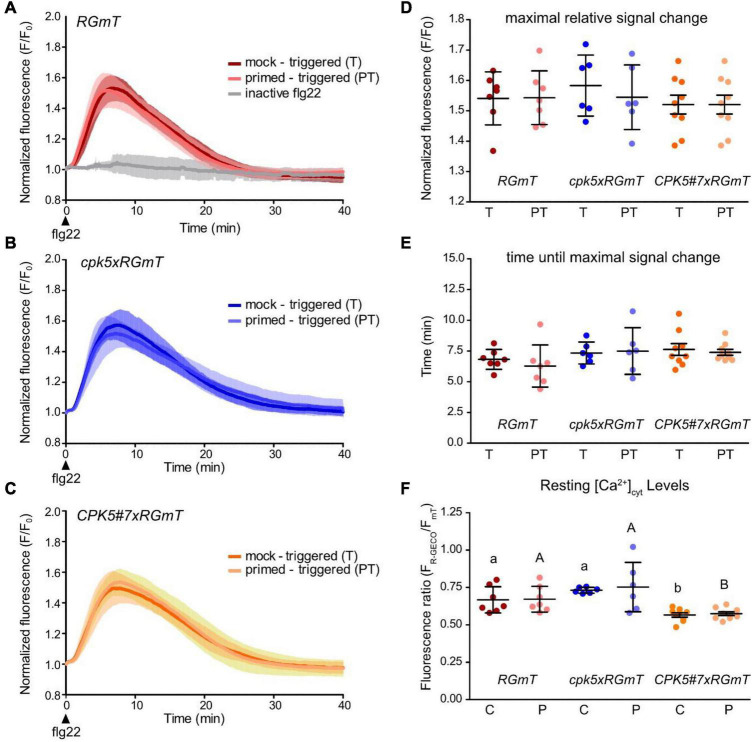
Defense priming has no significant effect on stimulus-induced calcium concentration changes in systemic tissue. **(A–C)** Flg22-induced calcium changes were recorded in systemic leaf disks 2 days after pre-treatment *via* infiltration of local leaves with 10 mM MgCl_2_ (mock) or 200 nM flg22 (primed). As triggering stimulus in the systemic leaf, 200 nM flg22 was applied, leading to the samples mock – triggered (T) or primed – triggered (PT), respectively. For detailed experimental setup, see Figure 4. As a control, inactive flg22 from *A. tumefaciens* was used as triggering stimulus with local mock pre-treatment. Data are averages of R-GECO1 fluorescence over time normalized to R-GECO1 fluorescence intensity at time point of flg22 application (t_0_). Error bars denote mean ± SD for *n* replicates (*RGmT n*_T_ = 7, *n*_*PT*_ = 7, *n*_inactive flg22_ = 3, **(B)**, *cpk5xRGmT n*_T_ = 6, *n*_*PT*_ = 6 and **(C)**, *CPK5#7xRGmT n*_T_ = 9, *n*_*PT*_ = 9). Parameters of flg22 induced calcium changes of measurements shown in panels **(A–C)** are analyzed in panels **(D–F)**. In panel **(D)** dot plots represent the amplitude of the maximal signal change, while in panel **(E)** the correlating time points of maximum signal after flg22 treatment are shown. Dots represent the individual measurements. Shown are means ± SD of 6 – 9 biological replicates per line for each treatment (T vs. PT). **(F)** Apparent resting F_R–GECO1_/F_*mT*_ in systemic leaf disks of local mock (10 mM MgCl_2_, control = C) or 200 nM flg22 (primed = P) treated plants. Shown are means ± SD (*n* ≥ 6). Dots represent the individual measurements. Two-way ANOVA and Bonferroni post test (*p* ≤ 0.05) reveals no significant differences between the pre-treatments but significant differences between genotypes indicated by different letters.

For comparing flg22-induced [Ca^2+^]_cyt_ changes in systemic leaf disks from primed vs. mock-treated plants, the pooled data of all measurements are summarized in Figure 5, with the individual Ca^2+^ traces of the 40 min recording period displayed in Supplementary Figure 5. The whole area of the leaf disk was quantified for a global overview of all cells within these disks. We have decided for this global overview analyses, in contrast to the analyses of selected smaller regions (as in Figure 1) for two reasons: (1) we cannot predict which cell has been “primed” for systemic immunity, and (2) it is known from our RGmT measurements that the flg22-induced response may not be synchronous (Figure 1 and Supplementary Figure 3).

In general, no significant differences were discernible that could be associated with the priming status of the plants, neither in wild type (Figure 5A), nor in the *cpk5* mutant (Figure 5B) or CPK5-OE lines (Figure 5C). More specifically, the quantitative data did not reveal any differences in the Ca^2+^ response in terms of the overall Ca^2+^ signature (Figures 5A–C), maximal fluorescence signal chance (Figure 5D) or peak response time (i.e., time after flg22 application until maximal signal chance) (Figure 5E). When assessing the basal resting [Ca^2+^]_cyt_, we observed an apparent lower value in CPK5#7 similar to what we have seen before in a local flg22 response in the absence of a pre-treatment. We therefore re-evaluated the resting [Ca^2+^]_cyt_ based on R-GECO1 imaging normalized to mT (fluorescence ratio) within our priming experiments and found that the resting [Ca^2+^]_cyt_ was with statistical significance lower in the CPK5-overexpressing line *CPK5#7xRGmT* compared to the wild-type or *cpk5* carrying RGmT (Figure 5F), irrespectively of a priming or a mock pre-treatment.

Taking our data from wild-type subjected to a priming and triggering treatment together with those obtained with the CPK5-OE line, which is already constitutively “primed” and capable of stimulus-dependent “super-priming,” these data indicate that the immune status of a plant has no (detectable) influence on a stimulus-induced Ca^2+^ response downstream of flg22 perception in systemic tissues under the imposed conditions.

## Discussion

“Priming” in plant immunity describes the state of a plant, in which a preceding primary infection by microbial pathogens induced an immune memory, so that the plant is prepared when challenged by subsequent infection. In SAR, the ability to display faster and stronger defense reactions is correlated with molecular changes of the plant characterized by distinct patterns of systemic defense signaling molecules (NHP), phytohormone levels (SA, JA) and expression of key transcription factors (*SARD1*) (Truman et al., 2007; Chen et al., 2018; Hartmann et al., 2018; Sun et al., 2018, 2020; Kim et al., 2020; Lim et al., 2020; Schnake et al., 2020; Vlot et al., 2021). Furthermore, these characteristic responses are often interconnected with each other in auto-activation and -synthesis loops. Our objective in this work is to investigate whether the intracellular Ca^2+^ change in response to a direct secondary, triggering stimulus is likewise altered as a consequence of its integration in such a systemic activation loop.

In the context of priming, we quantified the Ca^2+^ response in leaf disks of adult plants upon a triggering flg22 stimulus from plants that had or had not experienced a previous flg22 priming treatment. The leaf disk approach bears the opportunity to average signals over a multitude (>*n* 10^3^) of cells. While the aequorin-based system (Ranf et al., 2011) can similarly provide global Ca^2+^ response of multiple cells simultaneously, it is not suitable for our study because the seedlings commonly used are too young to mount a systemic immune memory. Furthermore, concurrent observation of multiple cells is of particular importance in SAR because it is unpredictable which and how many cells have undergone systemic priming and built an immune memory upon the pre-treatment 2 days earlier. Only these cells would give rise to priming-dependent [Ca^2+^] changes. To exclude that our chosen imaging approach may average out the individual cell responses if only a minority of cells react differently after priming, we included the CPK5-OE line in our studies. This line is characterized through molecular markers to be constitutively primed (Supplementary Figure 1) and capable of “super-priming” responses (Guerra et al., 2020). Additionally, averaging of single cell Ca^2+^ signals led successfully to the characterization of priming in response to cold stress (Knight et al., 1996; Knight and Knight, 2000).

By contrast, when comparing a local induced Ca^2+^ response in different genetic knock-out lines, Ca^2+^ changes can be traced in a few or even a single cell resolution, assuming that all cells are equally affected in a defined genetic background. Indeed, when selecting ROIs that cover only few cells, we observed distinct traces that displayed an oscillating Ca^2+^ pattern for approximately 30 min reminiscent to what has been reported by Keinath et al. (2015). While these distinct peaks became evident in selected ROIs covering single to few cells, an oscillatory Ca^2+^ pattern was less pronounced in the whole ROI image. These data validate the suitability of the R-GECO1 calcium sensor for investigating calcium changes in 6-week-old plants, required for priming experiments, and for the recording in epidermal peels combined with a bottom imaging setup. Under conditions of enhanced CPK5 signaling, more distinct Ca^2+^ peaks with slightly higher amplitudes could be recorded compared to the wild type and even more so compared to *cpk5* and *rbohd* mutants (Figures 1, 2 and Supplementary Figure 3). We have validated for the whole imaging ROI that the Ca^2+^ pattern was not affected by the number of imaged (47 ± 17) cells (Supplementary Figure 4). Therefore, the more distinct Ca^2+^ transients observed in the CPK5-OE lines may have a biological cause directly related to CPK5 activity itself. CPK5 is known to activate NADPH-oxidase RBOHD, and a Ca^2+^- and ROS-mediated defense signal propagation has been discussed (Dubiella et al., 2013). Our data suggest that upon flg22 stimulation, enhanced CPK5 activity leads to a reinforcement and local synchronization of Ca^2+^ signaling in these lines. Such interpretation is corroborated by the video depicting distinct (local) waves of Ca^2+^ signals induced by flg22 in the CPK5-OE line compared to *RGmT* (Supplementary Movies 1, 2). It is tempting to speculate that this reinforced Ca^2+^ signature mediated by CPK5 is not only part of the signal propagation to neighboring cells but is likewise responsible in each single cell to translate the Ca^2+^ signature into downstream defense reactions. In this context, it is worth to note that the whole image ROI shows fewer distinct peaks in the *rbohd* and *cpk5* mutants (Figure 2A). Likewise, the selected ROIs single cell traces also show fewer peaks in *cpk5* (Figure 2D). In support of the proposed amplificatory role of CPK5 in signal propagation (Dubiella et al., 2013; Guerra et al., 2020), statistically significant increase of the peak intervals is seen in the CPK5-OE (Figure 2F). Taken together, these data indicate that CPK5 may synchronize Ca^2+^ signaling within and between cells, and the absence of CPK5 leads to a reduction in both. RBOHD, which is biochemically phosphorylated and activated by CPK5, is required for the Ca^2+^ signal propagation to neighboring cells. This interpretation is consistent with our previous data showing that RBOHD and CPK5 constitute an auto-activating mechanism for defense signal spread to distal tissues (Dubiella et al., 2013). Here, we provide evidence that RBOHD contributes to the CPK5-reinforced synchronization for intercellular Ca^2+^ signal propagation.

How can the “oscillatory” Ca^2+^ signature in the CPK5-OE line be explained mechanistically? One possible explanation is a more coordinated in- and efflux of cytosolic Ca^2+^, i.e., activation of the Ca^2+^ efflux and inhibition of Ca^2+^ influx. Thus, CPK5 may contribute to the [Ca^2+^]_cyt_ homeostasis by promoting Ca^2+^ efflux out of the cytosol. Corresponding Ca^2+^ efflux transporters may be found among Ca^2+^/H^+^ exchangers (CAX) driven by electrochemical gradients of H^+^ and autoinhibited P-type II Ca^2+^-ATPases (ACA) (Sanders et al., 2002; Kudla et al., 2010; Bose et al., 2011; Demidchik et al., 2018). ACAs have been described in immune signaling (Boursiac et al., 2010; Frei dit Frey et al., 2012). Interestingly, the analysis of *aca4 aca11* mutants lacking ACA type of Ca^2+^ pumps revealed an increase in basal and flg22-induced rise in [Ca^2+^]_cyt_ (Hilleary et al., 2020). P-type II ACA- Ca^2+^-pumps are regulated by protein phosphorylation and some members are characterized by an N-terminal autoinhibition domain (Giacometti et al., 2012; Costa et al., 2017). Similarly, PAMP-responsive Ca^2+^-permeable channels are known to be regulated by phosphorylation (Tian et al., 2019; Thor et al., 2020). Whether such Ca^2+^ pumps and Ca^2+^ channels can be directly phosphorylated by CPK5 and contribute to the encoding of oscillations remains to be shown. It is noteworthy, in this context, that CPK5 displays a rather low *K*_*d*_ for Ca^2+^ of ∼100 nM for kinase activity. For other CDPKs auto-phosphorylation at its N-terminal domain can shift substrate accessibility (Ito et al., 2010, 2017, 2018). Such a mechanism could provide an additional layer in CPK5 regulation. Both mechanisms may render CPK5 as a suitable Ca^2+^ sensor-kinase effector-protein to reinforce “oscillatory” Ca^2+^ changes, which possibly include a Ca^2+^-induced Ca^2+^ release mechanism (Choi et al., 2014).

A natural follow-up question is: “How are the “more pronounced and defined number of Ca^2+^ peaks” observed in the CPK5-OE line decoded into enhanced basal defense responses within a cell?” Based on what is known from plant immune signaling in pattern-triggered, effector-triggered or systemic immunity in SAR, this will involve the myriad of different Ca^2+^-sensor, -relay and -effector proteins, such as CaM/CML, kinases or CAMTA transcription factors (Kudla et al., 2010). In the absence of additional evidence, one may further speculate that the decoding of Ca^2+^ transients into downstream defense responses may depend on the “counting” of encoded cytosolic (and/or nuclear) Ca^2+^ changes (up and downs in [Ca^2+^]) rather than a monophasic Ca^2+^ change.

In summary, our data provide evidence for an altered CPK5-dependent Ca^2+^ signature upon flg22 treatment in local tissues. This is consistent with CPK5 function as a predominant Ca^2+^ sensor and effector in pattern-triggered immunity mediating Ca^2+^ signal synchronization and defense response activation. However, the flg22-induced [Ca^2+^]_cyt_ increase in systemic cells are not different in the various priming context. Therefore, at least according to our experimental system, rapid Ca^2+^ changes in systemic tissues do not reflect the plant memory of “having been primed.”

## Data Availability Statement

The raw data supporting the conclusions of this article will be made available by the authors, without undue reservation.

## Author Contributions

BE, SL, JL, AL, and TR conceived and designed the strategy and experiments. BE, SL, and AL generated the RGmT lines. BE conducted all priming experiments and analyzed the systemic calcium imaging. SL and AL analyzed the local calcium imaging. FT conducted the aequorin-based calcium assays. XJ conducted the molecular characterization of RGmT lines. TG generated the aequorin lines. RW designed and generated the RGmT construct. BE, SL, XJ, AL, and JL prepared the figures. JL, AL, and TR wrote the manuscript. All authors discussed the results and commented on the manuscript.

## Conflict of Interest

The authors declare that the research was conducted in the absence of any commercial or financial relationships that could be construed as a potential conflict of interest.

## Publisher’s Note

All claims expressed in this article are solely those of the authors and do not necessarily represent those of their affiliated organizations, or those of the publisher, the editors and the reviewers. Any product that may be evaluated in this article, or claim that may be made by its manufacturer, is not guaranteed or endorsed by the publisher.

## References

[B1] Aldon D. Mbengue M. Mazars C. Galaud J. P. (2018). Calcium signalling in plant biotic interactions. *Int. J. Mol. Sci.* 19:665. 10.3390/ijms19030665 29495448 PMC5877526

[B2] Batistič O. Kudla J. (2012). Analysis of calcium signaling pathways in plants. *Biochim. Biophys. Acta* 1820 1283–1293. 10.1016/j.bbagen.2011.10.012 22061997

[B3] Blume B. Nürnberger T. Nass N. Scheel D. (2000). Receptor-mediated increase in cytoplasmic free calcium required for activation of pathogen defense in parsley. *Plant Cell* 12 1425–1440. 10.1105/tpc.12.8.1425 10948260 PMC149113

[B4] Bose J. Pottosin I. I. Shabala S. S. Palmgren M. G. Shabala S. (2011). Calcium efflux systems in stress signaling and adaptation in plants. *Front. Plant Sci.* 2:85. 10.3389/fpls.2011.00085 22639615 PMC3355617

[B5] Boudsocq M. Willmann M. R. McCormack M. Lee H. Shan L. He P. (2010). Differential innate immune signalling via Ca^2+^ sensor protein kinases. *Nature* 464 418–422. 10.1038/nature08794 20164835 PMC2841715

[B6] Boursiac Y. Lee S. M. Romanowsky S. Blank R. Sladek C. Chung W. S. (2010). Disruption of the vacuolar calcium-ATPases in *Arabidopsis* results in the activation of a salicylic acid-dependent programmed cell death pathway. *Plant Physiol.* 154 1158–1171. 10.1104/pp.110.159038 20837703 PMC2971596

[B7] Bredow M. Monaghan J. (2019). Regulation of plant immune signaling by calcium-dependent protein kinases. *Mol. Plant Microbe Interact.* 32 6–19. 10.1094/mpmi-09-18-0267-fi 30299213

[B8] Bredow M. Bender K. W. Johnson Dingee A. Holmes D. R. Thomson A. Ciren D. (2021). Phosphorylation-dependent subfunctionalization of the calcium-dependent protein kinase CPK28. *Proc. Natl. Acad. Sci. U.S.A.* 118:e2024272118. 10.1073/pnas.2024272118 33941701 PMC8126791

[B9] Chen Y. C. Holmes E. C. Rajniak J. Kim J. G. Tang S. Fischer C. R. (2018). N-hydroxy-pipecolic acid is a mobile metabolite that induces systemic disease resistance in *Arabidopsis*. *Proc. Natl. Acad. Sci. U.S.A.* 115 E4920–E4929. 10.1073/pnas.1805291115 29735713 PMC6003486

[B10] Choi W. G. Toyota M. Kim S. H. Hilleary R. Gilroy S. (2014). Salt stress-induced Ca^2+^ waves are associated with rapid, long-distance root-to-shoot signaling in plants. *Proc. Natl. Acad. Sci. U.S.A.* 111 6497–6502. 10.1073/pnas.1319955111 24706854 PMC4035928

[B11] Costa A. Luoni L. Marrano C. A. Hashimoto K. Köster P. Giacometti S. (2017). Ca^2+^-dependent phosphoregulation of the plasma membrane Ca^2+^-ATPase ACA8 modulates stimulus-induced calcium signatures. *J. Exp. Bot.* 68 3215–3230. 10.1093/jxb/erx162 28531251 PMC5853299

[B12] Czechowski T. Stitt M. Altmann T. Udvardi M. K. Scheible W. R. (2005). Genome-wide identification and testing of superior reference genes for transcript normalization in *Arabidopsis*. *Plant Physiol.* 139 5–17. 10.1104/pp.105.063743 16166256 PMC1203353

[B13] DeFalco T. A. Toyota M. Phan V. Karia P. Moeder W. Gilroy S. (2017). Using GCaMP3 to study Ca^2+^ signaling in *Nicotiana* species. *Plant Cell Physiol.* 58 1173–1184. 10.1093/pcp/pcx053 28482045

[B14] Demidchik V. Shabala S. Isayenkov S. Cuin T. A. Pottosin I. (2018). Calcium transport across plant membranes: mechanisms and functions. *New Phytol.* 220 49–69. 10.1111/nph.15266 29916203

[B15] Dodd A. N. Jakobsen M. K. Baker A. J. Telzerow A. Hou S. W. Laplaze L. (2006). Time of day modulates low-temperature Ca^2+^ signals in *Arabidopsis*. *Plant J.* 48 962–973. 10.1111/j.1365-313X.2006.02933.x 17227550

[B16] Dubiella U. Seybold H. Durian G. Komander E. Lassig R. Witte C. P. (2013). Calcium-dependent protein kinase/NADPH oxidase activation circuit is required for rapid defense signal propagation. *Proc. Natl. Acad. Sci. U.S.A.* 110 8744–8749. 10.1073/pnas.1221294110 23650383 PMC3666735

[B17] Fichman Y. Mittler R. (2021). Integration of electric, calcium, reactive oxygen species and hydraulic signals during rapid systemic signaling in plants. *Plant J.* 107 7–20. 10.1111/tpj.15360 34058040

[B18] Franz S. Ehlert B. Liese A. Kurth J. Cazalé A. C. Romeis T. (2011). Calcium-dependent protein kinase CPK21 functions in abiotic stress response in *Arabidopsis thaliana*. *Mol. Plant* 4 83–96. 10.1093/mp/ssq064 20978086

[B19] Frei dit Frey N. Mbengue M. Kwaaitaal M. Nitsch L. Altenbach D. Häweker H. (2012). Plasma membrane calcium ATPases are important components of receptor-mediated signaling in plant immune responses and development. *Plant Physiol.* 159 798–809. 10.1104/pp.111.192575 22535420 PMC3375942

[B20] Gao X. Chen X. Lin W. Chen S. Lu D. Niu Y. (2013). Bifurcation of *Arabidopsis* NLR immune signaling via Ca^2+^-dependent protein kinases. *PLoS Pathog.* 9:e1003127. 10.1371/journal.ppat.1003127 23382673 PMC3561149

[B21] Geiger D. Scherzer S. Mumm P. Marten I. Ache P. Matschi S. (2010). Guard cell anion channel SLAC1 is regulated by CDPK protein kinases with distinct Ca^2+^ affinities. *Proc. Natl. Acad. Sci. U.S.A.* 107 8023–8028. 10.1073/pnas.0912030107 20385816 PMC2867891

[B22] Giacometti S. Marrano C. A. Bonza M. C. Luoni L. Limonta M. De Michelis M. I. (2012). Phosphorylation of serine residues in the N-terminus modulates the activity of ACA8, a plasma membrane Ca^2+^-ATPase of *Arabidopsis thaliana*. *J. Exp. Bot.* 63 1215–1224. 10.1093/jxb/err346 22090438 PMC3276087

[B23] Guerra T. Schilling S. Hake K. Gorzolka K. Sylvester F. P. Conrads B. (2020). Calcium-dependent protein kinase 5 links calcium signaling with N-hydroxy-l-pipecolic acid- and SARD1-dependent immune memory in systemic acquired resistance. *New Phytol.* 225 310–325. 10.1111/nph.16147 31469917

[B24] Hake K. Romeis T. (2019). Protein kinase-mediated signalling in priming: immune signal initiation, propagation, and establishment of long-term pathogen resistance in plants. *Plant Cell Environ.* 42 904–917. 10.1111/pce.13429 30151921

[B25] Hartmann M. Zeier T. Bernsdorff F. Reichel-Deland V. Kim D. Hohmann M. (2018). Flavin monooxygenase-generated N-hydroxypipecolic acid is a critical element of plant systemic immunity. *Cell* 173 456–469.e416. 10.1016/j.cell.2018.02.049 29576453

[B26] Hilker M. Schmulling T. (2019). Stress priming, memory, and signalling in plants. *Plant Cell Environ.* 42 753–761. 10.1111/pce.13526 30779228

[B27] Hilleary R. Paez-Valencia J. Vens C. Toyota M. Palmgren M. Gilroy S. (2020). Tonoplast-localized Ca^2+^ pumps regulate Ca^2+^ signals during pattern-triggered immunity in *Arabidopsis thaliana*. *Proc. Natl. Acad. Sci. U.S.A.* 117 18849–18857. 10.1073/pnas.2004183117 32690691 PMC7414185

[B28] Ito T. Ishida S. Takahashi Y. (2018). Autophosphorylation of Ser-6 via an intermolecular mechanism is important for the rapid reduction of *Nt*CDPK1 kinase activity for substrate RSG. *PLoS One* 13:e0196357. 10.1371/journal.pone.0196357 29684069 PMC5912773

[B29] Ito T. Ishida S. Oe S. Fukazawa J. Takahashi Y. (2017). Autophosphorylation affects substrate-binding affinity of tobacco Ca^2+^-dependent protein kinase 1. *Plant Physiol.* 174:2457. 10.1104/pp.17.00515 28637832 PMC5543960

[B30] Ito T. Nakata M. Fukazawa J. Ishida S. Takahashi Y. (2010). Alteration of substrate specificity: the variable N-terminal domain of tobacco Ca^2+^-dependent protein kinase is important for substrate recognition. *Plant Cell* 22 1592–1604. 10.1105/tpc.109.073577 20442373 PMC2899867

[B31] Kadota Y. Sklenar J. Derbyshire P. Stransfeld L. Asai S. Ntoukakis V. (2014). Direct regulation of the NADPH oxidase RBOHD by the PRR-associated kinase BIK1 during plant immunity. *Mol. Cell* 54 43–55. 10.1016/j.molcel.2014.02.021 24630626

[B32] Keinath N. F. Waadt R. Brugman R. Schroeder J. I. Grossmann G. Schumacher K. (2015). Live cell imaging with R-GECO1 sheds light on flg22- and chitin-induced transient [Ca^2+^]_cyt_ patterns in *Arabidopsis*. *Mol. Plant* 8 1188–1200. 10.1016/j.molp.2015.05.006 26002145 PMC5134422

[B33] Kiep V. Vadassery J. Lattke J. Maaß J. P. Boland W. Peiter E. (2015). Systemic cytosolic Ca^2+^ elevation is activated upon wounding and herbivory in *Arabidopsis*. *New Phytol.* 207 996–1004. 10.1111/nph.13493 25996806

[B34] Kim Y. Gilmour S. J. Chao L. Park S. Thomashow M. F. (2020). *Arabidopsis* CAMTA transcription factors regulate pipecolic acid biosynthesis and priming of immunity genes. *Mol. Plant* 13 157–168. 10.1016/j.molp.2019.11.001 31733370

[B35] Knight H. Knight M. R. (2000). Imaging spatial and cellular characteristics of low temperature calcium signature after cold acclimation in *Arabidopsis*. *J. Exp. Bot.* 51 1679–1686. 10.1093/jexbot/51.351.1679 11053457

[B36] Knight H. Trewavas A. J. Knight M. R. (1996). Cold calcium signaling in *Arabidopsis* involves two cellular pools and a change in calcium signature after acclimation. *Plant Cell* 8 489–503. 10.1105/tpc.8.3.489 8721751 PMC161115

[B37] Knight M. R. Campbell A. K. Smith S. M. Trewavas A. J. (1991). Recombinant aequorin as a probe for cytosolic free Ca^2+^ in *Escherichia coli*. *FEBS Lett.* 282 405–408. 10.1016/0014-5793(91)80524-7 2037058

[B38] Kudla J. Batistic O. Hashimoto K. (2010). Calcium signals: the lead currency of plant information processing. *Plant Cell* 22 541–563. 10.1105/tpc.109.072686 20354197 PMC2861448

[B39] Kudla J. Becker D. Grill E. Hedrich R. Hippler M. Kummer U. (2018). Advances and current challenges in calcium signaling. *New Phytol.* 218 414–431. 10.1111/nph.14966 29332310

[B40] Kwaaitaal M. Huisman R. Maintz J. Reinstädler A. Panstruga R. (2011). Ionotropic glutamate receptor (iGluR)-like channels mediate MAMP-induced calcium influx in *Arabidopsis thaliana*. *Biochem. J.* 440 355–365. 10.1042/bj20111112 21848515

[B41] Lampropoulos A. Sutikovic Z. Wenzl C. Maegele I. Lohmann J. U. Forner J. (2013). GreenGate—a novel, versatile, and efficient cloning system for plant transgenesis. *PLoS One* 8:e83043. 10.1371/journal.pone.0083043 24376629 PMC3869738

[B42] Li K. Prada J. Damineli D. S. C. Liese A. Romeis T. Dandekar T. (2021). An optimized genetically encoded dual reporter for simultaneous ratio imaging of Ca^2+^ and H^+^ reveals new insights into ion signaling in plants. *New Phytol.* 230 2292–2310. 10.1111/nph.17202 33455006 PMC8383442

[B43] Liese A. Romeis T. (2013). Biochemical regulation of in vivo function of plant calcium-dependent protein kinases (CDPK). *Biochim. Biophys. Acta* 1833 1582–1589. 10.1016/j.bbamcr.2012.10.024 23123193

[B44] Lim G. H. Liu H. Yu K. Liu R. Shine M. B. Fernandez J. (2020). The plant cuticle regulates apoplastic transport of salicylic acid during systemic acquired resistance. *Sci. Adv.* 6:eaaz0478. 10.1126/sciadv.aaz0478 32494705 PMC7202870

[B45] Maintz J. Cavdar M. Tamborski J. Kwaaitaal M. Huisman R. Meesters C. (2014). Comparative analysis of MAMP-induced calcium influx in *Arabidopsis* seedlings and protoplasts. *Plant Cell Physiol.* 55 1813–1825. 10.1093/pcp/pcu112 25231962

[B46] McCormack E. Tsai Y. C. Braam J. (2005). Handling calcium signaling: *Arabidopsis* CaMs and CMLs. *Trends Plant Sci.* 10 383–389. 10.1016/j.tplants.2005.07.001 16023399

[B47] Mohanta T. K. Yadav D. Khan A. L. Hashem A. Abd Allah E. F. Al-Harrasi A. (2019). Molecular players of EF-hand containing calcium signaling event in plants. *Int. J. Mol. Sci.* 20:1476. 10.3390/ijms20061476 30909616 PMC6471108

[B48] Monaghan J. Matschi S. Romeis T. Zipfel C. (2015). The calcium-dependent protein kinase CPK28 negatively regulates the BIK1-mediated PAMP-induced calcium burst. *Plant Signal. Behav.* 10:e1018497. 10.1080/15592324.2015.1018497 26039480 PMC4622532

[B49] Monaghan J. Matschi S. Shorinola O. Rovenich H. Matei A. Segonzac C. (2014). The calcium-dependent protein kinase CPK28 buffers plant immunity and regulates BIK1 turnover. *Cell Host Microbe* 16 605–615. 10.1016/j.chom.2014.10.007 25525792

[B50] Nguyen C. T. Kurenda A. Stolz S. Chetelat A. Farmer E. E. (2018). Identification of cell populations necessary for leaf-to-leaf electrical signaling in a wounded plant. *Proc. Natl. Acad. Sci. U.S.A.* 115 10178–10183. 10.1073/pnas.1807049115 30228123 PMC6176584

[B51] R Core Team (2021). *R: A Language and Environment for Statistical Computing.* Vienna: Foundation for Statistical Computing.

[B52] Ranf S. Eschen-Lippold L. Pecher P. Lee J. Scheel D. (2011). Interplay between calcium signalling and early signalling elements during defence responses to microbe- or damage-associated molecular patterns. *Plant J.* 68 100–113. 10.1111/j.1365-313X.2011.04671.x 21668535

[B53] Ranf S. Grimmer J. Pöschl Y. Pecher P. Chinchilla D. Scheel D. (2012). Defense-related calcium signaling mutants uncovered via a quantitative high-throughput screen in *Arabidopsis thaliana*. *Mol. Plant* 5 115–130. 10.1093/mp/ssr064 21859959

[B54] Rentel M. C. Knight M. R. (2004). Oxidative stress-induced calcium signaling in *Arabidopsis*. *Plant Physiol.* 135 1471–1479. 10.1104/pp.104.042663 15247375 PMC519063

[B55] Romeis T. Herde M. (2014). From local to global: CDPKs in systemic defense signaling upon microbial and herbivore attack. *Curr. Opin. Plant Biol.* 20 1–10. 10.1016/j.pbi.2014.03.002 24681995

[B56] Sanders D. Pelloux J. Brownlee C. Harper J. F. (2002). Calcium at the crossroads of signaling. *Plant Cell* 14 S401–S417. 10.1105/tpc.002899 12045291 PMC151269

[B57] Schindelin J. Arganda-Carreras I. Frise E. Kaynig V. Longair M. Pietzsch T. (2012). Fiji: an open-source platform for biological-image analysis. *Nat. Methods* 9 676–682. 10.1038/nmeth.2019 22743772 PMC3855844

[B58] Schmittgen T. D. Livak K. J. (2008). Analyzing real-time PCR data by the comparative C_T_ method. *Nat. Protoc.* 3 1101–1108. 10.1038/nprot.2008.73 18546601

[B59] Schnake A. Hartmann M. Schreiber S. Malik J. Brahmann L. Yildiz I. (2020). Inducible biosynthesis and immune function of the systemic acquired resistance inducer N-hydroxypipecolic acid in monocotyledonous and dicotyledonous plants. *J. Exp. Bot.* 71 6444–6459. 10.1093/jxb/eraa317 32725118 PMC7586749

[B60] Schulz P. Herde M. Romeis T. (2013). Calcium-dependent protein kinases: hubs in plant stress signaling and development. *Plant Physiol.* 163 523–530. 10.1104/pp.113.222539 24014579 PMC3793034

[B61] Seybold H. Trempel F. Ranf S. Scheel D. Romeis T. Lee J. (2014). Ca^2+^ signalling in plant immune response: from pattern recognition receptors to Ca^2+^ decoding mechanisms. *New Phytol.* 204 782–790. 10.1111/nph.13031 25539002

[B62] Shao Q. Gao Q. Lhamo D. Zhang H. Luan S. (2020). Two glutamate- and pH-regulated Ca^2+^ channels are required for systemic wound signaling in *Arabidopsis*. *Sci. Signal.* 13:eaba1453. 10.1126/scisignal.aba1453 32665412

[B63] Shi S. Li S. Asim M. Mao J. Xu D. Ullah Z. (2018). The *Arabidopsis* calcium-dependent protein kinases (CDPKs) and their roles in plant growth regulation and abiotic stress responses. *Int. J. Mol. Sci.* 19:1900. 10.3390/ijms19071900 29958430 PMC6073581

[B64] Simeunovic A. Mair A. Wurzinger B. Teige M. (2016). Know where your clients are: subcellular localization and targets of calcium-dependent protein kinases. *J. Exp. Bot.* 67 3855–3872. 10.1093/jxb/erw157 27117335

[B65] Sun T. Busta L. Zhang Q. Ding P. Jetter R. Zhang Y. (2018). TGACG-BINDING FACTOR 1 (TGA1) and TGA4 regulate salicylic acid and pipecolic acid biosynthesis by modulating the expression of *SYSTEMIC ACQUIRED RESISTANCE DEFICIENT 1* (*SARD1*) and *CALMODULIN-BINDING PROTEIN 60g* (*CBP60g*). *New Phytol.* 217 344–354. 10.1111/nph.14780 28898429

[B66] Sun T. Huang J. Xu Y. Verma V. Jing B. Sun Y. (2020). Redundant CAMTA transcription factors negatively regulate the biosynthesis of salicylic acid and N-hydroxypipecolic acid by modulating the expression of *SARD1* and *CBP60g*. *Mol. Plant* 13 144–156. 10.1016/j.molp.2019.10.016 31733371

[B67] Thor K. Peiter E. (2014). Cytosolic calcium signals elicited by the pathogen-associated molecular pattern flg22 in stomatal guard cells are of an oscillatory nature. *New Phytol.* 204 873–881. 10.1111/nph.13064 25243759

[B68] Thor K. Jiang S. Michard E. George J. Scherzer S. Huang S. (2020). The calcium-permeable channel OSCA1.3 regulates plant stomatal immunity. *Nature* 585 569–573. 10.1038/s41586-020-2702-1 32846426 PMC8435934

[B69] Tian W. Hou C. Ren Z. Wang C. Zhao F. Dahlbeck D. (2019). A calmodulin-gated calcium channel links pathogen patterns to plant immunity. *Nature* 572 131–135. 10.1038/s41586-019-1413-y 31316205

[B70] Tian W. Wang C. Gao Q. Li L. Luan S. (2020). Calcium spikes, waves and oscillations in plant development and biotic interactions. *Nat. Plants* 6 750–759. 10.1038/s41477-020-0667-6 32601423

[B71] Torres M. A. Dangl J. L. Jones J. D. (2002). *Arabidopsis* gp91phox homologues *At*rbohD and *At*rbohF are required for accumulation of reactive oxygen intermediates in the plant defense response. *Proc. Natl. Acad. Sci. U.S.A.* 99 517–522. 10.1073/pnas.012452499 11756663 PMC117592

[B72] Toyota M. Spencer D. Sawai-Toyota S. Jiaqi W. Zhang T. Koo A. J. (2018). Glutamate triggers long-distance, calcium-based plant defense signaling. *Science* 361 1112–1115. 10.1126/science.aat7744 30213912

[B73] Trempel F. Ranf S. Scheel D. Lee J. (2016). Quantitative analysis of microbe-associated molecular pattern (MAMP)-induced Ca^2+^ transients in plants. *Methods Mol. Biol.* 1398 331–344. 10.1007/978-1-4939-3356-3_27 26867636

[B74] Truman W. Bennett M. H. Kubigsteltig I. Turnbull C. Grant M. (2007). *Arabidopsis* systemic immunity uses conserved defense signaling pathways and is mediated by jasmonates. *Proc. Natl. Acad. Sci. U.S.A.* 104 1075–1080. 10.1073/pnas.0605423104 17215350 PMC1783366

[B75] Vlot A. C. Sales J. H. Lenk M. Bauer K. Brambilla A. Sommer A. (2021). Systemic propagation of immunity in plants. *New Phytol.* 229 1234–1250. 10.1111/nph.16953 32978988

[B76] Waadt R. Krebs M. Kudla J. Schumacher K. (2017). Multiparameter imaging of calcium and abscisic acid and high-resolution quantitative calcium measurements using R-GECO1-mTurquoise in *Arabidopsis*. *New Phytol.* 216 303–320. 10.1111/nph.14706 28850185

[B77] Wickham H. (2007). Reshaping data with the reshape package. *J. Stat. Softw.* 21 1–20. 10.18637/jss.v021.i12

[B78] Wickham H. Averick M. Bryan J. Chang W. D’Agostino McGowan L. François R. (2019). Welcome to the Tidyverse. *J. Open Source Softw.* 43:1686. 10.21105/joss.01686

[B79] Yip Delormel T. Boudsocq M. (2019). Properties and functions of calcium-dependent protein kinases and their relatives in *Arabidopsis thaliana*. *New Phytol.* 224 585–604. 10.1111/nph.16088 31369160

