# Supplementary material for: Plant Immune Memory in Systemic Tissue Does Not Involve Changes in Rapid Calcium Signaling
Source: Front Plant Sci. 2021 Dec 14;12:798230. doi: 10.3389/fpls.2021.798230 (PMC8712724; doi:10.3389/fpls.2021.798230)
Supplement: Supplementary file 1 [file Data_Sheet_1.pdf]

Supplementary Material

(for Eichstädt et al.; contains 3 supplementary movies, 6 supplementary figures and 1 supplementary table)

1 Supplementary Data

Movie S1. Flg22-Induced Calcium Transients in Leaf Epidermal Cells of *RGmT* Plants (Wild-type Background). Time lapse movie of R-GECO1-based Ca^{2+} imaging in leaf epidermal peels of 6-week-old plants of *RGmT* line. Treatment with 200 nM flg22 at time point 10 minutes. Movie corresponds to an exemplary measurement taken from data shown in Figure 1B, S3. Scale bar, 100 µm. Time format, mm:ss.

Movie S2. Flg22-Induced Calcium Transients in Leaf Epidermal Cells of *CPK5#7xRGmT* line. Time lapse movie of R-GECO1-based Ca^{2+} imaging in leaf epidermal peels of 6-week-old plants of *CPK5#7xRGmT* line. Treatment with 200 nM flg22 at time point 10 minutes. Movie corresponds to an exemplary measurement taken from data shown in Figure 1B, S3. Scale bar, 100 µm. Time format, mm:ss.

Movie S3. Comparison of Flg22-Induced Calcium Transients in Systemic Leaf of Mock-treated or Primed Plants. Time lapse movie of R-GECO1-based Ca^{2+} imaging were recorded in systemic leaf discs of *RGmT* line 2 days after pre-treatment via infiltration of local leaves with 10 mM $MgCl_2$ (mock) or 200 nM flg22 (primed). As triggering stimulus in the systemic leaf, 200 nM flg22 was used, leading to the samples mock – triggered (T, left part of the movie) or primed – triggered (PT, right part of the movie), respectively. Movie corresponds to exemplary measurements taken from data shown in Figure 5A, S5. Scale bar, 1 mm. Time format, mm:ss.

2 Supplementary Figures and Tables

2.1 Supplementary Figures

FIGURE S1

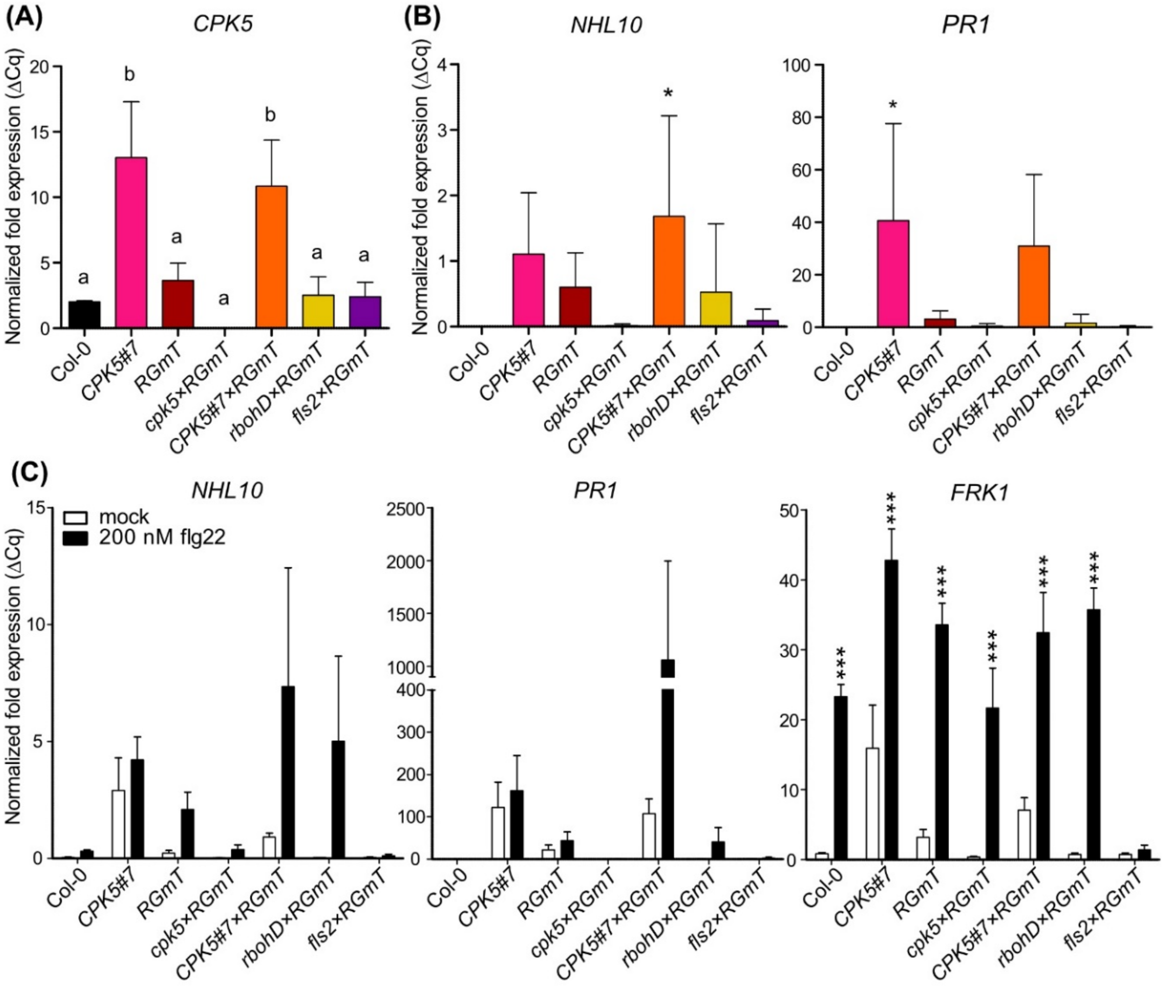

Figure S1: Basal and flg22-Induced Gene Expression of Defence Marker Genes in Calcium Sensor Line *RGmT* and Derived Crossing with *CPK5#7, cpk5, rbohD* and *fls2*.

Gene expression analysis in 6-week-old plants under basal conditions (A, B) and after flg22 treatment (C). (A) *CPK5* transcript analyses in indicated genotypes. Statistical analyses were conducted using one-way ANOVA and Tukey's multiple comparison test, $p \leq 0.05$. Different letters indicate significant differences between the genotypes. (B) Basal gene expression of the defence marker genes *NHL10* and *PR1*. One-way ANOVA and Dunnett's multiple comparison test reveal significant differences * = $p \leq 0.05$ to wild-type. (C) Data for *NHL10, PR1* and *FRK1* transcript levels after 3 h infiltration with 200 nM flg22 (black bars) or water as control (mock, white bars). Statistical differences were tested using two-way ANOVA and Bonferroni multiple comparison test, significant differences are indicated by *** = $p \leq 0.001$ between control and flg22 treatment. Values represent mean ± standard deviations (SD) of 4 biological replicates.

FIGURE S2

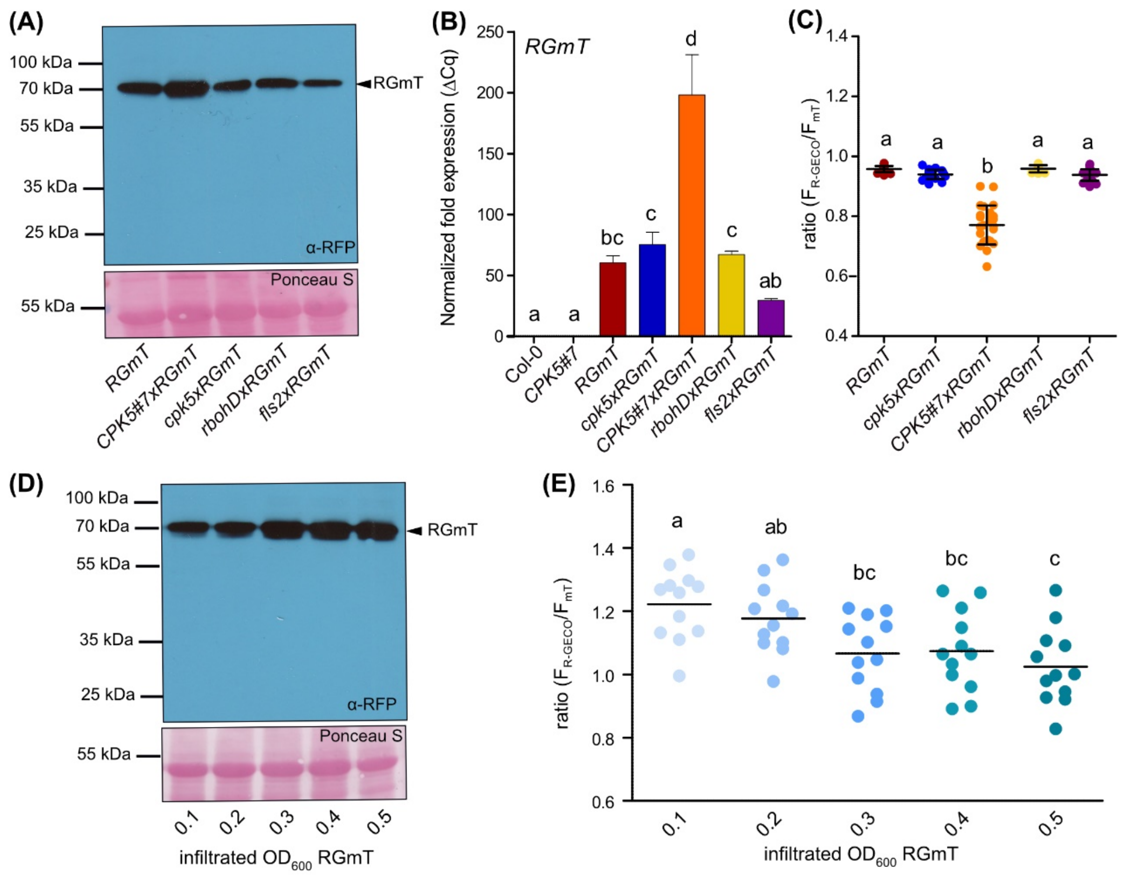

Figure S2: Calcium Sensor Concentration Influences Apparent Resting $F_{R\text{-}GECO1}/F_{mT}$ Ratios

(A) RGmT calcium sensor protein expression levels in *RGmT* and *RGmT* crosses with *cpk5*, *CPK5#7*, *rbohD*, and *fls2*. Protein crude extracts were separated by SDS gel electrophoresis and visualized by immunodetection using an anti-RFP detection system (upper panel). Ponceau S stained RuBisCO protein served as a loading control (lower panel). (B) Transcript levels of *RGmT*. Bars represent means ± SD of 4 biological replicates. Significant differences marked by different letters (one-way ANOVA and Tukey's multiple comparison test, $p \leq 0.05$). (C) $F_{R\text{-}GECO1}/F_{mT}$ ratios in *RGmT* and derived crosses with *cpk5*, CPK5 #7, *rbohD*, and *fls2*. Dot plot indicates the fluorescence ratio (R) of R-GECO1 and mTurquoise of leaf discs ($n \geq 18$). Dots represent the individual measurements. Shown are means ± SD. Different letters denote groups with significant different means (One-way ANOVA, Tukey's multiple comparison test, $p \leq 0.05$). The experiment was repeated three times with similar results. (D) Protein expression levels of RGmT expressed in *N. benthamiana*. Crude protein extracts of leaf discs were separated by SDS gel electrophoresis and visualized by immunodetection using an anti-RFP detection system (upper panel). Ponceau S stained RuBisCO protein served as a loading control (lower panel). (E) Fluorescence ratio (R) of R-GECO1 and mTurquoise after transient expression in *Nicotiana benthamiana*. Agrobacteria carrying RGmT coding plasmid with an OD_{600} range between 0.1 and 0.5 were used to express a protein concentration gradient. Dots represent the individual measurements. Significant differences are marked by different letters (one-way ANOVA and Tukey's multiple comparison test, $p \leq 0.05$).

FIGURE S3

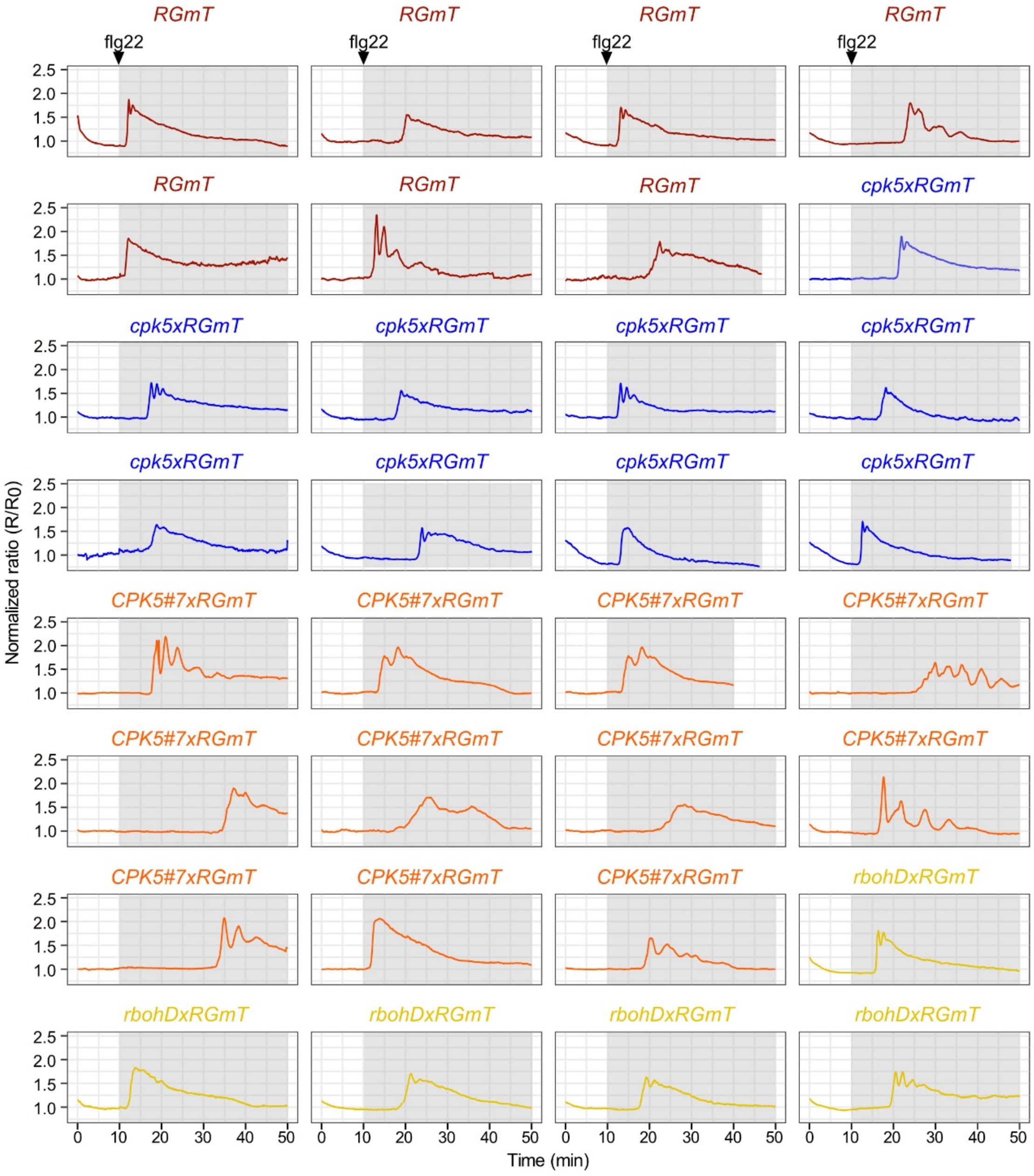

Figure S3: Additional Single Traces of Calcium Changes in Response to flg22 in Epidermal Cells of Mutant and Overexpression Lines (Supplemental to Figure 1).

Normalized $F_{R\text{-}GECO1}/F_{mT}$ ratios (R/R_0) after flg22 treatment calculated from total acquired image (ROI_1) of epidermal peels from 6-week-old plants. Results from 5 to 11 independent biological replicates per RGmT carrying lines derived from crossing are shown. These are additional measurements related to data presented in Figure 1. The 40 min time interval of recording after application of the 200 nM flg22 treatment is indicated by the underlying gray area.

Figure S4

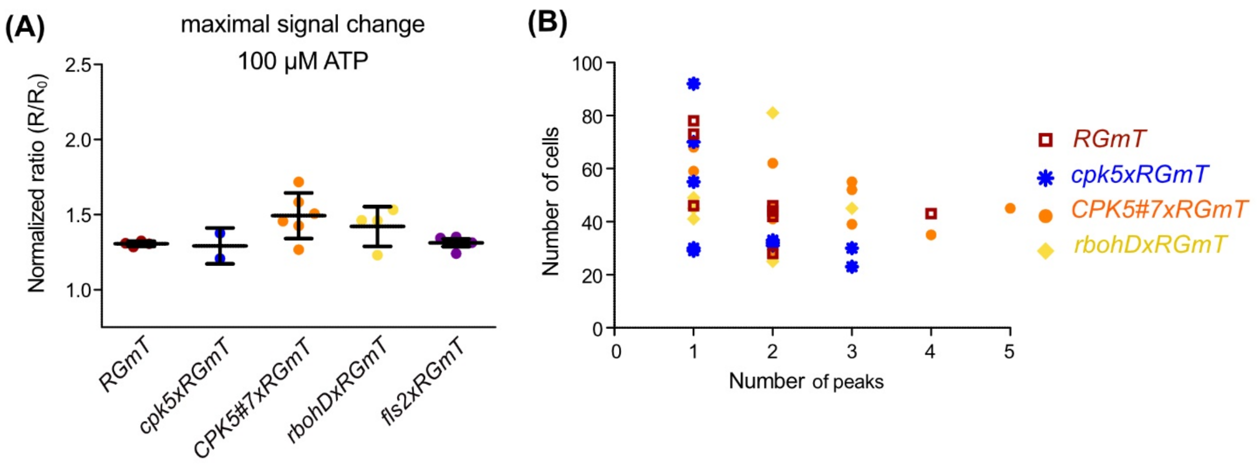

Figure S4: ATP-Induced Local Calcium Transients and Analysis for Correlation between Number of Cells Imaged per Area to Observed Number of Peaks.

(A) Scatter plot of maximal signal change after treatment with 100 µM ATP. Shown are means ± SD of 2-6 biological replicates from three independent sets. Dots represent the individual measurements. One-way ANOVA ($p \leq 0.05$) reveals no significant differences over all genotypes. (B) To check if the observed peak numbers may be biased by cell numbers within the imaged area, the number of cells in the whole image ROI (ROI_1) were plotted over the number of peaks after local flg22 treatment from the indicated genotypes ($n \geq 6$ biological replicates). Dots represent the individual measurements of the flg22-induced calcium transients shown in Figure 1 and Figure S3. Linear regression of all measurements reveals no significant deviation of the slope from 0, so that differential cell numbers (resulting from genotype or plant size) are not consequential for the observed peak numbers.

FIGURE S5

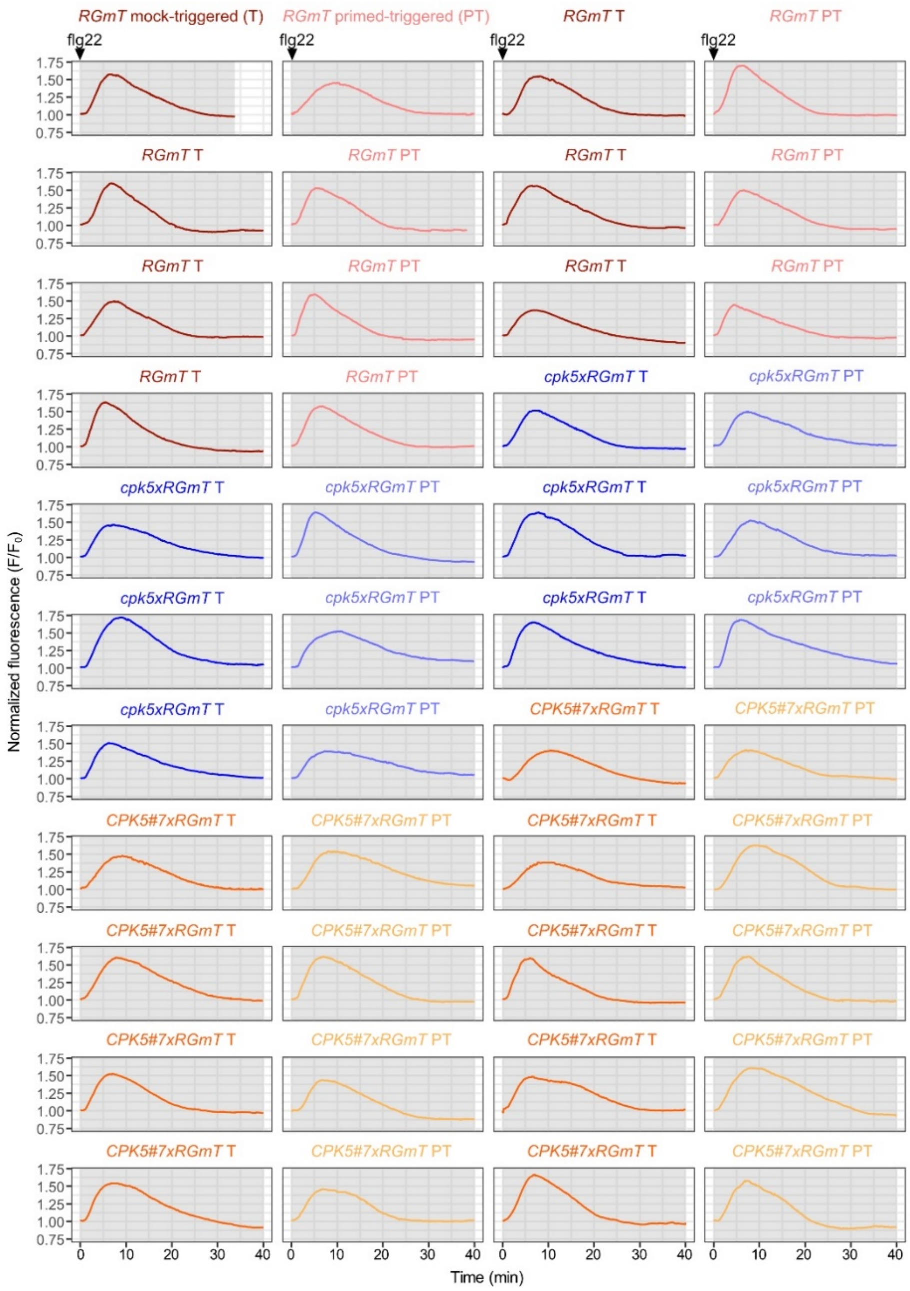

Figure S5: Single Traces of Calcium Changes in Response to flg22 in Systemic Tissue of *R-GECO1-mTurquoise* (*RGmT*) expressing lines

Flg22-induced calcium changes were recorded in systemic leaf discs 2 days after pre-treatment via infiltration of local leaves with 10 mM $MgCl_2$ (mock) or 200 nM flg22 (primed) in the indicated genotypes. For detailed experimental setup, see Figure 4. Shown are the individual normalized R-GECO1 fluorescence traces of the systemic leaf exposed to 200 nM flg22 of the "mock – triggered (T)" or "primed – triggered (PT)" samples.

FIGURE S6

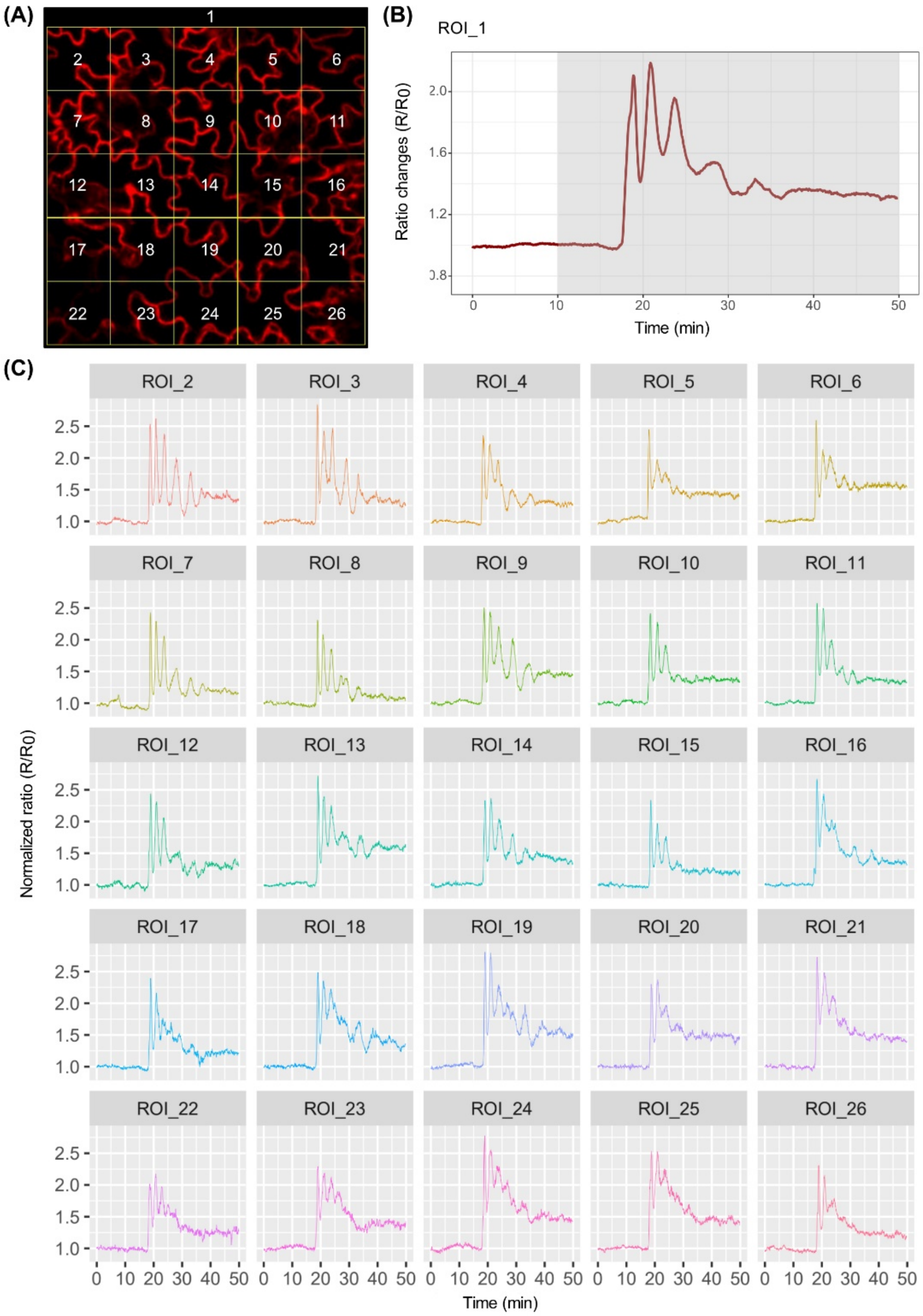

Figure S6: Workflow of Calcium Pattern Analyses in Response to flg22 in Epidermal Cells

For the analysis of the local calcium changes in response to flg22, the acquired image was subdivided into 25 ROIs leading to the whole image ROI_1 and 25 smaller ROIs (ROI_2-ROI_26). The selected ROIs are shown for one exemplary measurement in (A). ROI_1 is defined as the whole acquired image. For the generation of the ROIs, an ImageJ script was used to generate the same ROIs for all measurements (see materials and methods). (B, C) Single traces of the normalized $F_{R\text{-}GECO1}/F_{mT}$ ratios in ROI_1 (B) and all generated sections ROI_2 – ROI_26 (C). These graphs were generated for all measurements using a custom-made R-script.

Table S1: Sequences of RT-qPCR oligonucleotide primers

AtCPK5-fw-qRT	TAACAGCGGGGCAATCACAT
AtCPK5-rev-qRT	GTCTACATCAGCCGCATCCA
R-Geco 1-fw-qRT	GACTACACCATCGTGGAACAGT
R-Geco 1-rev-qRT	TTATCCTCCTCGCCCTTGCT
NHL10-fw-qRT	ACGCCGGACAGTCTAGGA
NHL10-rev-qRT	CCCTAAGCCTGAACTTGATCTC
PR1-fw-qRT	TGATCCTCGTGGGAATTATGT
PR1-rev-qRT	TGCATGATCACATCATTACTTCAT
FRK1-fw-qRT	GAGACTATTTGGCAGGTAAAAGGT
FRK1-rev-qRT	AGGAGGCTTACAACCATTGTG
